# Metabolites of Medicarpin and Their Distributions in Rats

**DOI:** 10.3390/molecules24101966

**Published:** 2019-05-22

**Authors:** Hong-Yan Wang, Teng Li, Rui Ji, Feng Xu, Guang-Xue Liu, Yao-Li Li, Ming-Ying Shang, Shao-Qing Cai

**Affiliations:** 1 State Key Laboratory of Natural and Biomimetic Drugs, School of Pharmaceutical Sciences, Peking University, No. 38, Xueyuan Road, Beijing 100191, China; wanghongyan@bjmu.edu.cn (H.-Y.W.); tengli1175@163.com (T.L.); jiruiwonder@163.com (R.J.); guangxl@bjmu.edu.cn (G.-X.L.); liyaoli123456@163.com (Y.-L.L.); sqcai@bjmu.edu.cn (S.-Q.C.); 2 School of Pharmacy, Heilongjiang University of Chinese Medicine, No.24, Heping Road, Xiangfang District, Harbin 150040, China

**Keywords:** medicarpin, pterocarpan, metabolites, distribution, in vivo, HPLC-ESI-IT-TOF-MS^n^

## Abstract

Medicarpin is a bioactive pterocarpan that has been attracting increasing attention in recent years. However, its metabolic fate in vivo is still unknown. To clarify its metabolism and the distribution of its metabolites in rats after oral administration, the HPLC-ESI-IT-TOF-MS^n^ technique was used. A total of 165 new metabolites (13 phase I and 152 phase II metabolites) were tentatively identified, and 104, 29, 38, 41, 74, 28, 24, 15, 42, 8, 10, 3, and 17 metabolites were identified in urine, feces, plasma, the colon, intestine, stomach, liver, spleen, kidney, lung, heart, brain, and thymus, respectively. Metabolic reactions included demethylation, hydrogenation, hydroxylation, glucuronidation, sulfation, methylation, glycosylation, and vitamin C conjugation. **M1** (medicarpin glucuronide), **M5** (vestitol-1’-*O*-glucuronide) were distributed to 10 organs, and **M1** was the most abundant metabolite in seven organs. Moreover, we found that isomerization of medicarpin must occur in vivo. At least 93 metabolites were regarded as potential new compounds by retrieving information from the Scifinder database. This is the first detailed report on the metabolism of ptercarpans in animals, which will help to deepen the understanding of the metabolism characteristics of medicarpin in vivo and provide a solid basis for further studies on the metabolism of other pterocarpans in animals.

## 1. Introduction

Pterocarpans are the second largest group of dihydroisoflavonoids and have a special structure skeleton (benzopyran-benzofuran four-ring system). They were originally isolated from plants [1] and then regarded as an important group of phytoalexins, which have antifungal [2] and antibacterial [3] effects. In recent years, many new bioactivities of pterocarpans have been discovered, such as anti-inflammation [4,5,6], antitumor activity [7,8,9], anti-osteoporosis [10,11,12], antimalarial [13], antioxidant [14,15,16], inhibition of neuraminidase [17,18,19] and melanin synthesis [20], estrogenic and anti-estrogenic activity [21], anti-clastogensis [22], immunosuppressive activity [23], and inhibition of acetylcholinesterase [24]. As a result, this group of natural products is getting more and more attention. However, after conducting a systematic literature survey, we surprisingly found that there is only one report on the metabolism of a pure pterocarpan (i.e., trifolirhizin) in animals and only two metabolites (maackiain in feces and maackiain, maackiain-3-*O*-sulfoacid in urine) were identified [25], though a lot of papers about biosynthesis and the fungal metabolism of pterocarpans have been published. Drug metabolism research has a pivotal role in new drug research and development. In order to effectively utilize pterocarpans, it is important to clarify their metabolism characteristics.

**Figure 1:**
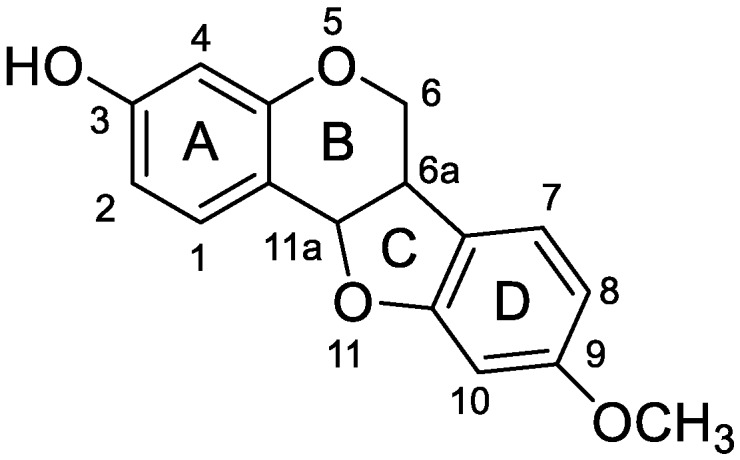
The structure and atom numbering of medicarpin.

Medicarpin (Figure 1) is a natural pterocarpan-type phytoalexin [26] with a wide variety of biological activities distributed in lots of traditional Chinese medicines (TCM), such as Pueraria Lobata [27], and Hedysari Radix [28]. According to the literature, medicarpin has anti-osteoporotic and bone protection activities even at a low concentration of 10^−4^ μM on murine bone cells and it can reduce the formation of osteoclasts but increase the formation of osteoprogenitor cells in bone marrow cells (BMCs) in OVx mice at a dose of 10 mg/kg/d for 30 days [10]. In another study, medicarpin treatment (10 mg/kg/d for 30 days) can increase the formation of osteoprogenitor cells in BMCs and osteoid formation in female Sprague-Dawley (SD) rats [11]. It also shows cytotoxic activities in BCA-1 (human breast cancer cells, IC_50_ = 13.14 μg/mL) and KB (human epidermoid carcinoma of cavity, IC_50_ = 10.13 μg/mL) cells [29]. In addition, medicarpin can lead to apoptosis and reverse multidrug resistance in P388 leukemia cells (IC_50_ ≈ 90 μM) [30]. It also has antifungal [31] and antibacterial activities [32]. Additionally, medicarpin can stimulate mesenchymal stem cells to differentiate into brown and beige adipocytes via the adenosine monophosphate-activated protein kinase (AMPK) pathway and boost lipolysis in adipocytes via a protein kinase A (PKA)-dependent pathway, so it is expected to be a potential drug for the treatment of obesity [33,34]. However, the metabolism of medicarpin in vivo and in vitro is still unknown. Therefore, the present study aims to identify its metabolites, clarify its metabolic pathways, and reveal the distribution of medicarpin and its metabolites in rats using high-performance liquid chromatography-electrospray ionization-ion trap-time of flight-multistage mass spectrometry (HPLC-ESI-IT-TOF-MS^n^).

## 2. Results and Discussion

### 2.1. MS Fragmentation Characteristics of Medicarpin in ESI− Mode and Identification of Medicarpin in Rats

**Figure 2:**
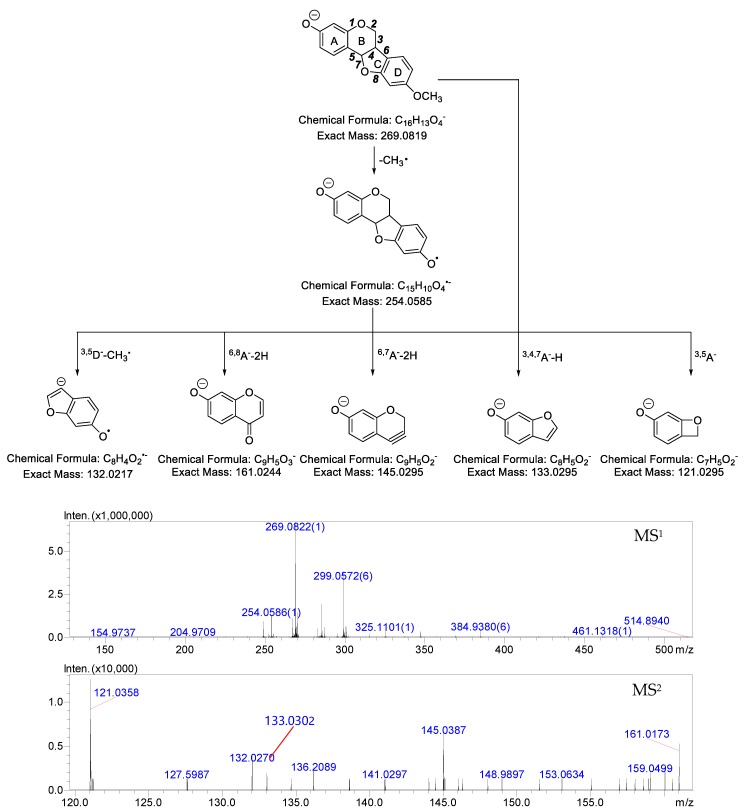
The MS spectra and the proposed fragmentation pathways of medicarpin in ESI− mode.

In order to facilitate the description of the ESI− MS characteristics of medicarpin and its metabolites, we proposed a nomenclature for the fragmentation pathways and fragment ions. The four rings were named A, B, C and D, respectively. The cleavable C-C bonds in the skeleton were designated by numbers 1–8, as shown in Figure 2.

Medicarpin (C_16_H_14_O_4_) showed [M − H]^−^ at *m*/*z* 269.0822 (theoretical mass was 269.0819 Da) in ESI− spectra. The characteristic fragment ions of medicarpin in ESI− mode included *m*/*z* 254.0586 ([M − H − CH_3_•]^•−^), *m*/*z* 161.0173 (C_9_H_5_O_3_, ^6,8^A^−^ − 2H), *m*/*z* 145.0387 (C_9_H_5_O_2_, ^6,7^A^−^ − 2H), *m*/*z* 133.0302 (C_8_H_5_O_2_, ^3,4,7^A^−^ − H), *m*/*z* 132.0270 (C_8_H_4_O_2_, ^3,5^D^−^ − CH_3_•), *m*/*z* 121.0358 (C_7_H_5_O_2_, ^3,5^A^−^) in its MS^2^ spectra (Figure 2).

### 2.2. Profiling 165 Metabolites of Medicarpin in Rats

The metabolites of medicarpin were screened by comparing the HPLC chromatograms and MS base peak chromatograms (BPCs) of drug and blank group samples obtained through HPLC-ESI-IT-TOF-MS^n^ analysis, and then confirmed by comparison of extracted ion chromatograms (EICs) between the drug and blank groups. The metabolic reactions were judged by the accurate mass (elemental composition) differences between medicarpin and its metabolites. The accurate mass (elemental composition) differences of −14.01 Da (CH_2_), +14.01 Da (CH_2_), +2.01 Da (H_2_), and +15.99 Da (O) indicated demethylation, methylation, hydrogenation and hydroxylation. The loss of 176.03 Da (C_6_H_8_O_6_) from precursor ion and/or an anion at *m*/*z* 175.02 in MS^2^ spectra indicated that the metabolite was a glucuronide; the loss of 79.95 Da (SO_3_) from precursor ion indicated that the metabolite was a sulfate; the loss of 162.05 Da (C_6_H_10_O_5_) indicated a hexoside (more likely to be a glucoside); the loss of 158.02 Da (C_6_H_6_O_5_) indicated a vitamin C conjugate [35].

**Figure 3:**
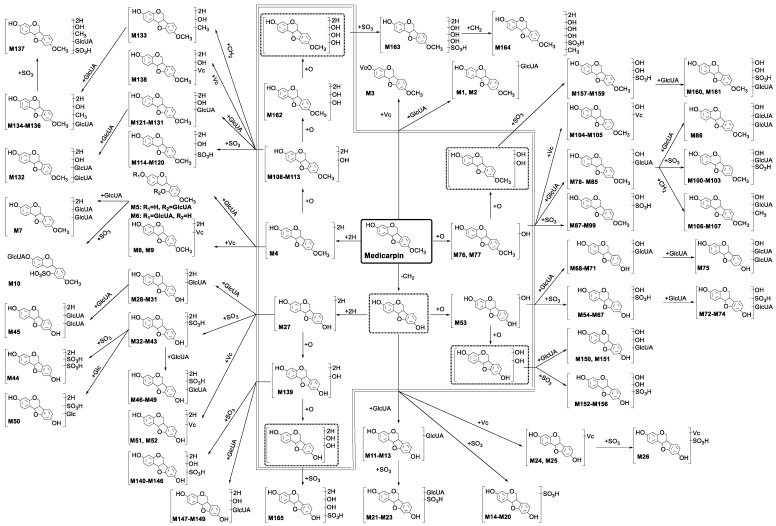
The proposed metabolic pathways of medicarpin in rats. Phase I metabolites are in the double line polygon, undetected intermediates are in the dashed line boxes.

A total of 165 metabolites of medicarpin were confirmed and tentatively identified by the above-mentioned method, and the proposed metabolic pathways of medicarpin are shown in Figure 3. According to the skeleton of molecules, 165 metabolites were classified into 13 categories: (1) metabolites (**M1**–**M3**) having the skeleton of medicarpin; (2) metabolites (**M4**–**M10**) having the skeleton of hydrogenated medicarpin; (3) metabolites (**M11**–**M26**) having the skeleton of demethylated medicarpin; (4) metabolites (**M27**–**M52**) having the skeleton of demethylated and hydrogenated medicarpin; (5) metabolites (**M53**–**M75**) having the skeleton of demethylated and hydroxylated medicarpin; (6) metabolites (**M76**–**M107**) having the skeleton of hydroxylated medicarpin; (7) metabolites (**M108**–**M138**) having the skeleton of hydrogenated and hydroxylated medicarpin; (8) metabolites (**M139**–**M149**) having the skeleton of demethylated, hydrogenated and hydroxylated medicarpin; (9) metabolites (**M150**–**M156**) having the skeleton of demethylated and dihydroxylated medicarpin; (10) metabolites (**M157**–**M161**) having the skeleton of dihydroxylated medicarpin; (11) metabolite (**M162**) having the skeleton of hydrogenated and dihydroxylated medicarpin; (12) metabolites (**M163**–**M164**) having the skeleton of hydrogenated and trihydroxylated medicarpin; (13) metabolite (**M165**) having the skeleton of demethylated, hydrogenated and dihydroxylated medicarpin. The EICs of 165 metabolites are shown in Figures S1–S44.

#### 2.2.1. Analysis of Metabolites (**M1**–**M3**) Having the Skeleton of Medicarpin

**Figure 4:**
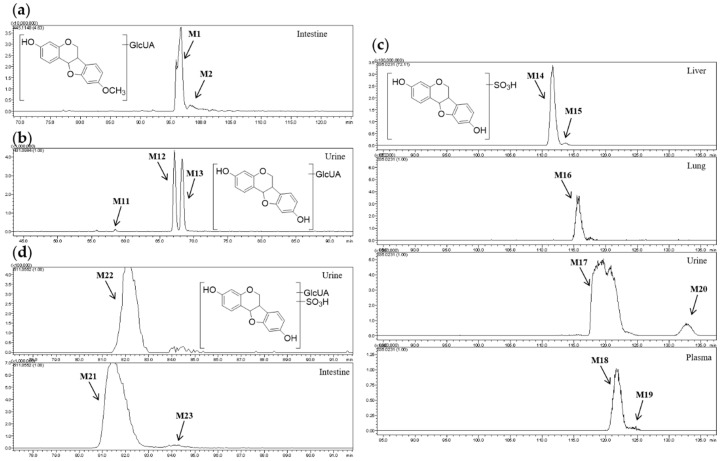
(**a**) Extracted ion chromatograms (EIC) of *m*/*z* 445.11; (**b**) EIC of *m*/*z* 431.10; (**c**) EIC of *m*/*z* 335.02; (**d**) EIC of *m*/*z* 511.06.

**M1** and **M2** showed [M − H]^−^ at *m*/*z* 445.11 and their molecular formulae were predicted to be C_22_H_22_O_10_, so they were isomers. The fragment ions at *m*/*z* 269.08 and *m*/*z* 175.02 can be detected in both of their MS^2^ spectra. Therefore, **M1** and **M2** were determined to be glucuronides of medicarpin. However, medicarpin only has one hydroxyl group, i.e., one glucuronidation site (Figure 4a). Hence, we deduced that isomerization of medicarpin (configuration change or shift of substituent) must occur.

**Figure 5:**
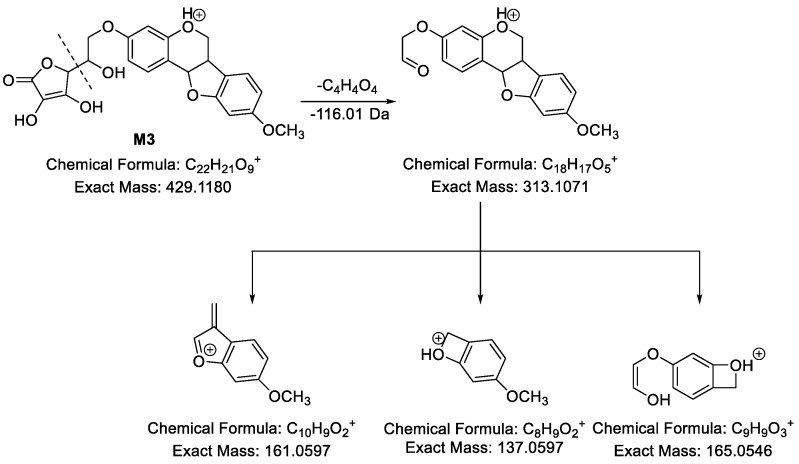
The proposed fragmentation pathways of **M3** in ESI+ mode.

**M3** (Figure 5) showed [M − H]^−^ at *m*/*z* 427.1049 and its molecular formula was predicted to be C_22_H_20_O_9_. In its MS^2^ spectra, *m*/*z* 269.0792 (C_16_H_1__3_O_4_, [medicarpin − H]^−^) formed by losing 158.02 Da (C_6_H_6_O_5_) from [M − H]^−^ was observed, indicating a vitamin C conjugate based on our previous study [35]. Besides, in the ESI+ mode, **M3** showed [M + H]^+^ at *m*/*z* 429.1177. In its MS^2^ spectra, fragment ion at *m*/*z* 313.1113 was observed, indicating a neutral loss of 116.01 Da (C_4_H_4_O_4_). Subsequently, *m*/*z* 313.1113 yielded fragment ions at *m*/*z* 165.0516 (C_9_H_9_O_3_), *m*/*z* 161.0577 (C_10_H_9_O_2_) and *m*/*z* 137.0600 (C_8_H_9_O_2_). Hence, **M3** was medicarpin-3-*O*-vitamin C.

#### 2.2.2. Analysis of Metabolites (**M4**–**M10**) Having the Skeleton of Hydrogenated Medicarpin

**Figure 6:**
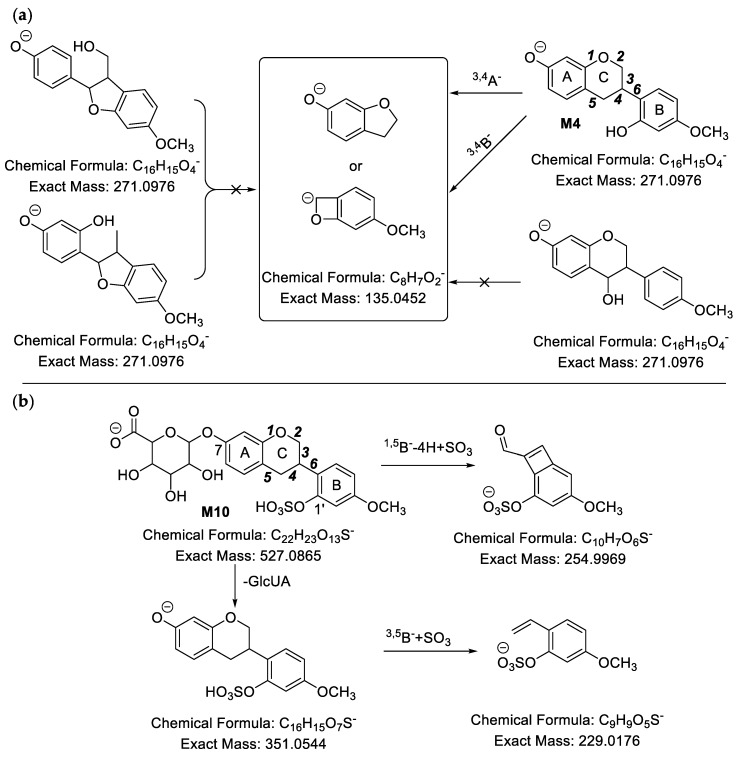
(**a**) The proposed fragmentation pathways of **M4** in ESI− mode; (**b**) The proposed fragmentation pathways of **M10** in ESI− mode.

**M4** showed [M − H]^−^ at *m*/*z* 271.0979, and its molecular formula was predicted to be C_16_H_16_O_4_, which had two more hydrogen atoms than that of medicarpin. Therefore, one ring of medicarpin must be cleaved. In its MS^2^ spectra, the fragment ions at *m*/*z* 147.0469 (C_9_H_7_O_2_, ^6^A^−^ or ^3,5^B^−^ − 2H ), *m*/*z* 135.0466 (C_8_H_7_O_2_, ^3,4^A^−^ or ^3,4^B^−^), *m*/*z* 123.0543 (C_7_H_7_O_2_, ^6^B^−^ + H or ^3,5^A^−^ + 2H or ^2,4^A^−^ + 2H), *m*/*z* 121.0302 (C_7_H_5_O_2_, ^3,5^A^−^ or ^2,4^A^−^) and *m*/*z* 109.0321 (C_6_H_5_O_2_, ^2,5^A^−^ + 2H) were detected. According to the characteristic fragment ion at *m*/*z* 135.0466 (C_8_H_7_O_2_, ^3,4^A^−^ or ^3,4^B^−^), we could deduce that medicarpin had undergone ring cleavage at the bond of C11-C11a as shown in Figure 6a. Hence, **M4** was determined to be vestitol. The characteristic fragment ion at *m*/*z* 135.05 (C_8_H_7_O_2_) was very useful, because its existence indicated that the C11-C11a bond was cleaved and the hydrogenated medicarpin had the skeleton of a vestitol. The MS^2^ spectrum of **M4** is shown in Figure S45.

**M5** and **M6** showed [M − H]^−^ at *m*/*z* 447.13, and their molecular formulae were predicted to be C_22_H_24_O_10_. Moreover, the fragment ions at *m*/*z* 271.09, *m*/*z* 175.02, *m*/*z* 147.05 (C_9_H_7_O_2_, ^6^A^−^ or ^3,5^B^−^ − 2H) and *m*/*z* 135.05 (C_8_H_7_O_2_, ^3,4^A^−^ or ^3,4^B^−^) could be detected in their MS^2^ spectra. Based on *m*/*z* 135.05 (C_8_H_7_O_2_), we could deduce that the ring-cleavage took place at the bond of C11-C11a. Because a larger CLogP value means a lower polarity and a larger retention time in reversed phase HPLC, so **M5** (CLogP = 0.7229, t_R_ = 90.033) was assigned as vestitol-1’-*O*-glucuronide and **M6** (CLogP = 0.9229, t_R_ = 92.050) was vestitol-7-*O*-glucuronide.

**M8** and **M9** showed [M − H]^−^ at *m*/*z* 429.12 and their molecular formulae were predicted to be C_22_H_22_O_9_, and they yielded a fragment ion at *m*/*z* 271.09 by neutral loss of 158.02 Da (C_6_H_6_O_5_). In their MS^3^ spectra, the characteristic fragment ions at *m*/*z* 147.05 (C_9_H_7_O_2_, ^6^A^−^ or ^3,5^B^−^ − 2H), *m*/*z* 135.05 (C_8_H_7_O_2_, ^3,4^A^−^ or ^3,4^B^−^) and *m*/*z* 109.03 (C_6_H_5_O_2_, ^2,5^A^−^ + 2H) were observed. Thus, **M8** and **M9** were determined as vestitol vitamin C conjugates.

The molecular formula of **M10** (Figure 6b) was calculated as C_22_H_24_O_13_S based on the [M − H]^−^ at *m*/*z* 527.0883. The fragment ions at *m*/*z* 351.0505 and *m*/*z* 271.0944 were observed in MS^2^ spectra and *m*/*z* 147.0546 (C_9_H_7_O_2_), *m*/*z* 135.0498 (C_8_H_7_O_2_, ^3,4^A^−^ or ^3,4^B^−^) and *m*/*z* 121.0354 (C_7_H_5_O_2_) were detected in MS^3^ spectra. This indicated that **M10** was a hydrogenated medicarpin glucuronide sulfate. Besides, based on *m*/*z* 135.0498 (C_8_H_7_O_2_), we could deduce that the bond of C11-C11a in medicarpin was cleaved. Moreover, the fragment ion at *m*/*z* 254.9829 (C_10_H_7_O_6_S,), which was derived from precursor ion *m*/*z* 527.0901, indicated that the sulfonic group (-SO_3_H) was linked to the fragment of C_10_H_7_O_3_ (^1,5^B^−^ − 4H), and the fragment ion at *m*/*z* 229.0156 (C_9_H_9_O_5_S), which was obtained from precursor ion *m*/*z* 351.0505, indicated that -SO_3_H was linked to the fragment of C_9_H_9_O_2_ (^3,5^B^−^). Hence, we could deduce that the sulfonic group was linked to the hydroxyl group of B ring, while the glucuronyl group was linked to the hydroxyl group of A ring. Therefore, **M10** was unambiguously identified as vestitol-7-*O*-glucuronide-1’-*O*-sulfate. The MS spectra of **M10** are shown in Figure S46.

#### 2.2.3. Analysis of Metabolites (**M11**–**M26**) Having the Skeleton of Demethylated Medicarpin

**M11**–**M13** (Figure 4b) showed [M − H]^−^ at *m*/*z* 431.10, and their molecular formulae were predicted to be C_21_H_20_O_10_. Compared with [Aglycon − H]^−^ (C_15_H_11_O_4_) at *m*/*z* 255.07, the neutral loss was 176.03 Da (C_6_H_8_O_6_), indicating that **M11**–**M13** were glucuronides of demethylated medicarpin.

**M14**–**M20** (Figure 4c) were isomers whose molecular formulae were calculated to be C_15_H_12_O_7_S based on [M − H]^−^ at *m*/*z* 335.02. According to the neutral loss 79.95 Da (SO_3_), we speculated that **M14**–**M20** were sulfates of demethylated medicarpin.

The molecular formulae of **M21**–**M23** (Figure 4d) were predicted to be C_21_H_20_O_13_S based on [M − H]^−^ at *m*/*z* 511.06. In their MS^2^ spectra, fragment ions at *m*/*z* 335.02 and *m*/*z* 255.07 which were formed by sequential neutral losses of 176.03 Da (C_6_H_8_O_6_) and 79.95 Da (SO_3_) were observed, demonstrating that **M21**–**M23** were demethylated medicarpin glucuronide sulfates.

Demethylated medicarpin only has two metabolism sites (two free hydroxyl groups), but it has three glucuronidation metabolites (**M11**–**M13**), seven sulfation metabolites (**M14**–**M20**) and three glucuronidation and sulfation metabolites (**M21**–**M23**). Therefore, we can confirm that isomerization of medicarpin (configuration change or shift of substituent) must occur.

#### 2.2.4. Analysis of Metabolites (**M27**–**M52**) Having the Skeleton of Demethylated and Hydrogenated Medicarpin

**Figure 7:**
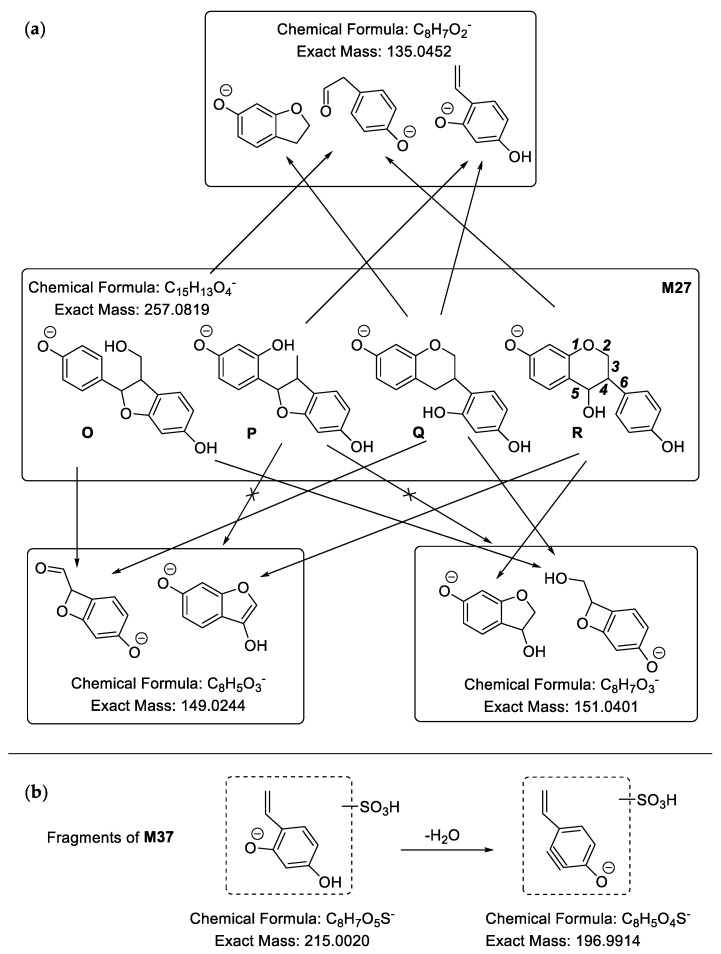
Characteristic fragment ions of **M27** (**a**) and **M37** (**b**) in ESI− mode.

**M27** (Figure 7a) showed [M − H]^−^ at *m*/*z* 257.0820, and the molecular formula was predicted to be C_15_H_14_O_4_. Compared with C_16_H_14_O_4_ of medicarpin, it has one less carbon atom. In its MS^2^ spectra, there were a series of fragment ions at *m*/*z* 149.0289 (C_8_H_5_O_3_), *m*/*z* 147.0481 (C_9_H_7_O_2_), *m*/*z* 135.0494 (C_8_H_7_O_2_), *m*/*z* 121.0329 (C_7_H_5_O_2_) and *m*/*z* 109.0263 (C_6_H_5_O_2_). Based on the fragment ion at 149.0289 (C_8_H_5_O_3_), we could determine that the **M27** might be **O**, **Q** or **R**. **M37** (Figure 7b) presented [M − H]^−^ at *m*/*z* 337.0392, therefore its molecular formula was calculated to be C_15_H_14_O_7_S. We could speculate that it was the sulfate of demethylated and hydrogenated medicarpin based on the neutral loss of 79.95 Da (SO_3_) and fragment ion at *m*/*z* 257.0804 ([Aglycon − H]^−^). Besides, fragment ions at *m*/*z* 151.0443 (C_8_H_7_O_3_), *m*/*z* 147.0456 (C_9_H_7_O_2_), *m*/*z* 135.0461 (C_8_H_7_O_2_), *m*/*z* 121.0267 (C_7_H_5_O_2_), *m*/*z* 119.0586 (C_8_H_7_O) and *m*/*z* 109.0234 (C_6_H_5_O_2_) were observed. Based on fragment ions at *m*/*z* 215.0033 (C_8_H_7_O_5_S) and *m*/*z* 196.9928 (C_8_H_5_O_4_S), which were yielded from *m*/*z* 215.0033 (C_8_H_7_O_5_S) by the loss of 18.01 Da (H_2_O), we could deduce that the skeleton of C_8_H_7_O_2_ had two hydroxyl groups and the sulfonic group was linked to the B ring. As a result, the aglycon of **M37** must be **Q**. The characteristic fragment ions of **M27** and **M37** are shown in Figure 7. The MS spectrum of **M37** is shown in Figure S47.

#### 2.2.5. Analysis of Metabolites (**M53**–**M75**) Having the Skeleton of Demethylated and Hydroxylated Medicarpin

**M53** presented [M − H]^−^ at *m*/*z* 271.0612, and the molecular formula was indicated to be C_15_H_12_O_5_. Compared with C_16_H_14_O_4_ of medicarpin, it has a less CH_2_ and one more oxygen atom. Furthermore, according to the fragment ions at *m*/*z* 161.0281 (C_9_H_5_O_3_), *m*/*z* 149.0273 (C_8_H_6_O_3_), *m*/*z* 137.0283 (C_7_H_5_O_3_), *m*/*z* 133.0249 (C_8_H_5_O_2_), *m*/*z* 121.0406 (C_7_H_5_O_2_) and *m*/*z* 109.0327 (C_6_H_5_O_2_), **M53** was determined to be demethylated and hydroxylated medicarpin. However, due to the special structure of the demethylated medicarpin, it is impossible to judge the position of hydroxylation based on the current mass spectrometry data.

#### 2.2.6. Analysis of Metabolites (**M76**–**M107**) Having the Skeleton of Hydroxylated Medicarpin

The molecular formulae of **M87**–**M99** were predicted to be C_16_H_14_O_8_S due to the pseudomolecular ion peak [M − H]^−^ at *m*/*z* 365.04. In their MS^2^ spectra, the fragment ion at *m*/*z* 285.08 (C_16_H_1__3_O_5_) formed by the loss of 79.95 Da (SO_3_) was detected. Therefore, **M87**–**M99** were hydroxylated medicarpin sulfates.

**Figure 8:**
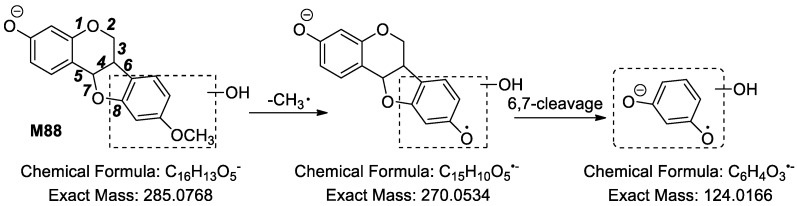
The proposed fragmentation pathways of **M88** in ESI− mode.

**M88** (Figure 8) possessed fragment ions at *m*/*z* 285.0727, *m*/*z* 270.0564, *m*/*z* 161.0254 (C_9_H_5_O_3_, ^6,8^A^−^ − 2H or ^3,5^B^−^ − 2H), *m*/*z* 139.0337 (C_7_H_7_O_3_, ^6,7^B^−^ + 2H) and *m*/*z* 124.0179 (C_6_H_4_O_3_, ^6,7^B^−^ + 2H − CH_3_•). Based on the fragment ion at *m*/*z* 124.0179 (C_6_H_4_O_3_, ^6,7^B^−^ + 2H − CH_3_•), generated from the fragment ion at *m*/*z* 270.0564, we deduced that the newly added hydroxyl group was linked to the D ring of **M88**.

#### 2.2.7. Analysis of Metabolites (**M108**–**M138**) Having the Skeleton of Hydrogenated and Hydroxylated Medicarpin

**Figure 9:**
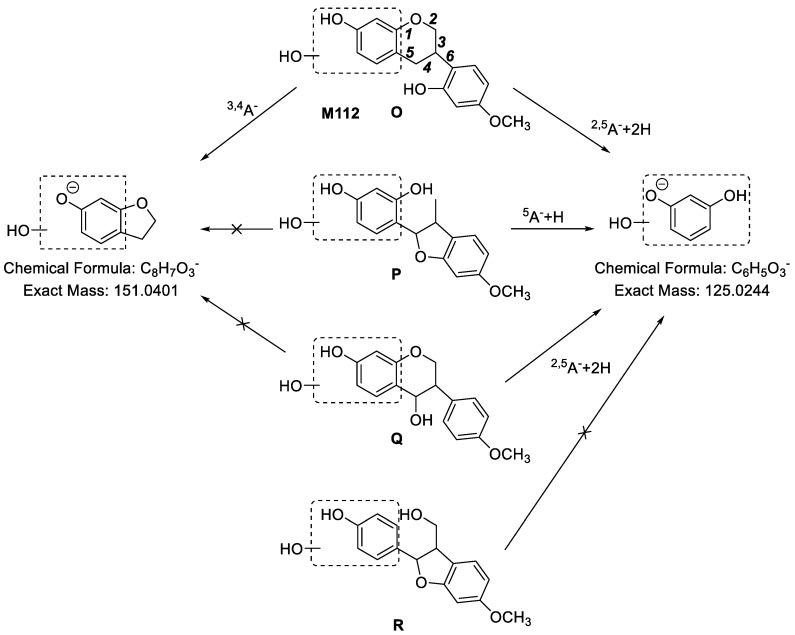
The proposed fragmentation pathways of **M112** in ESI− mode.

**M112** (Figure 9) showed [M − H]^−^ at *m*/*z* 287.0943 and the molecular formula was calculated to be C_16_H_16_O_5_, which was two hydrogen atoms and one oxygen atom more than that of medicarpin. Therefore, we speculated that **M112** was hydrogenated and hydroxylated medicarpin. In its MS^2^ spectra, a characteristic fragment ion at *m*/*z* 125.0181 (C_6_H_5_O_3_, ^2,5^A^−^ + 2H) was observed, indicating that the newly added hydroxyl group was linked to the A ring, so it has three possibilities **O**, **P** or **Q**. Besides, based on the fragment ion at *m*/*z* 151.0451 (C_8_H_7_O_3_, ^3,4^A^−^), we could determine that the bond of C11-C11a was cleaved. Finally, the structure of **M112** was determined to be **O**. Furthermore, the fragment ions of **M112** including *m*/*z* 163.0388 (C_9_H_7_O_3_, ^6^A^−^ − H), *m*/*z* 149.0532 (C_9_H_9_O_2_, ^3,5^B^−^), *m*/*z* 145.0348 (C_9_H_5_O_2_, ^6^A^−^ − H − H_2_O), *m*/*z* 137.0334 (C_7_H_5_O_3_, ^2,4^A^−^ or ^3,5^A^−^) and *m*/*z* 134.0482 (C_8_H_6_O_2_, ^3,5^B^−^ − CH_3_•) supported our elucidation. The MS^2^ spectrum of **M112** is shown in Figure S48.

The molecular formulae of **M114**–**M120** were predicted as C_16_H_16_O_8_S based on their [M − H]^−^ at *m*/*z* 367.05. In their MS^2^ spectra, *m*/*z* 287.09 (C_16_H_15_O_5_) was detected, indicating a neutral loss of 79.95 Da (SO_3_). Therefore, **M114**–**M120** were determined as hydrogenated and hydroxylated medicarpin sulfates.

**Figure 10:**
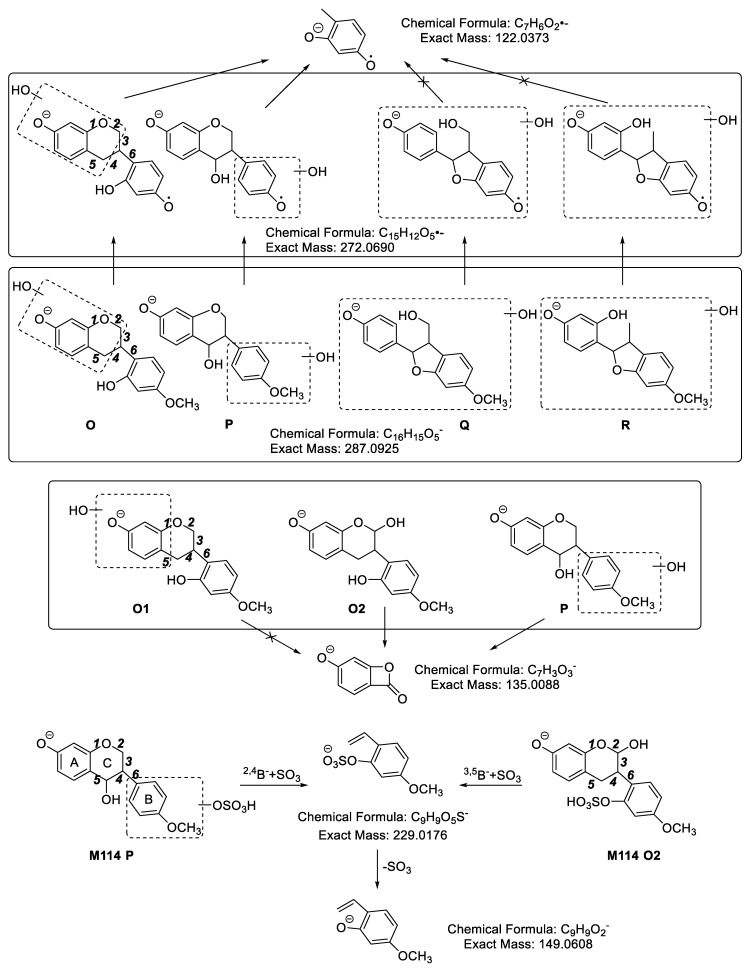
The proposed fragmentation pathways of **M114**.

**M114** (Figure 10) showed fragment ions at *m*/*z* 287.0921 ([Aglycon − H]^−^), *m*/*z* 272.0681 ([Aglycon − H]^−^ − CH_3_•), *m*/*z* 245.0126 (C_9_H_9_O_6_S), *m*/*z* 229.0108 (C_9_H_9_O_5_S), *m*/*z* 177.0598 (C_10_H_9_O_3_), *m*/*z* 165.0603 (C_9_H_9_O_3_), *m*/*z* 163.0388 (C_9_H_7_O_3_), *m*/*z* 161.0254 (C_9_H_5_O_3_), *m*/*z* 151.0417 (C_8_H_7_O_3_,), *m*/*z* 150.0382 (C_8_H_6_O_3_), *m*/*z* 149.0653 (C_9_H_9_O_2_), 137.0213 (C_7_H_5_O_3_), *m*/*z* 135.0589 (C_8_H_7_O_2_), *m*/*z* 135.0094 (C_7_H_3_O_3_), *m*/*z* 122.0312 (C_7_H_6_O_2_) and *m*/*z* 121.0363 (C_7_H_5_O_2_) in MS^2^ spectra. According to the fragment ion at *m*/*z* 122.0312 (C_7_H_6_O_2_), produced from *m*/*z* 272.0681 ([Aglycon − H]^−^ − CH_3_•), we determined that the aglycon of **M114** should be **O** or **P**. Moreover, based on the fragment ion at *m*/*z* 135.0094 (C_7_H_3_O_3_), we could deduce that the aglycon of **M114** was **O2** or **P**. However, because the same fragment ions could be produced from **O2** or **P**, it was impossible to judge the position of hydroxylation based on the current mass spectrometry data. In addition, the elemental composition of *m*/*z* 229.0108 (C_9_H_9_O_5_S) suggested that the sulfonic group was connected to the fragment ion of *m*/*z* 149.0653 (C_9_H_9_O_2_). Hence, we could determine the sulfate was linked to the hydroxyl group of B ring. The MS^2^ spectrum of **M114** is shown in Figure S49.

**Figure 11:**
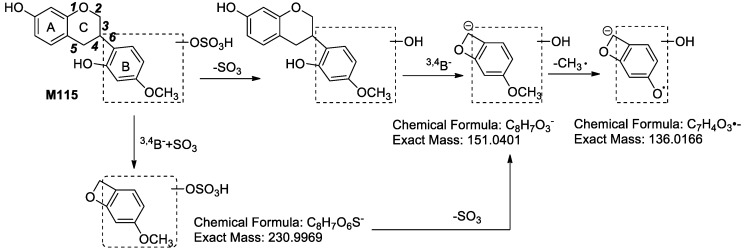
The probable structure and characteristic fragment ions of **M115**.

**M115** exhibited fragment ions at *m*/*z* 287.0918 (C_16_H_15_O_5_, [Aglycon − H]^−^), *m*/*z* 230.9941 (C_8_H_7_O_6_S, ^3,4^B^−^ + SO_3_), *m*/*z* 177.0590 (C_10_H_9_O_3_, ^2,5^B^−^ − 2H), *m*/*z* 165.0631 (C_9_H_9_O_3_, ^3,5^B^−^ or ^2,4^B^−^), *m*/*z* 151.0440 (C_8_H_7_O_3_, ^3,4^B^−^), *m*/*z* 150.0407 (C_9_H_9_O_3_, ^3,5^B^−^ − CH_3_• or ^2,4^B^−^ − CH_3_•), *m*/*z* 147.0412 (C_9_H_7_O_2_, ^6^A^−^ − H), *m*/*z* 139.0448 (C_7_H_7_O_3_, ^6^B^−^ + H), *m*/*z* 136.0182 (C_7_H_4_O_3_, ^3,4^B^−^ − CH_3_•), *m*/*z* 135.0397 (C_8_H_7_O_2_, ^3,4^A^−^) and *m*/*z* 121.0325 (C_7_H_5_O_2_, ^3,5^A^−^ or ^2,4^A^−^) in its MS^2^ spectra. From the characteristic fragment ion at *m*/*z* 136.0182 (C_7_H_4_O_3_, ^3,4^B^−^ − CH_3_•) yielded from *m*/*z* 151.0440 (C_8_H_7_O_3_, ^3,4^B^−^), we could identify that the newly added hydroxyl group was linked to the D ring of medicarpin and the C11-C11a bond of medicarpin was cleaved. Moreover, *m*/*z* 230.9941 was predicted to be C_8_H_7_O_6_S, which indicated that the sulfonic group was connected to the fragment ion of *m*/*z* 151.0440 (C_8_H_7_O_3_, ^3,4^B^−^). We therefore confirmed that the sulfonic group was linked to the hydroxyl group of B ring. The probable structure and characteristic fragment ions of **M115** are shown in Figure 11. The MS^2^ spectrum of **M115** is shown in Figure S50.

By comparing the fragmentation pathways of **M112**, **M114** and **M115**, we get the following information: (1) As long as the fragment ion at *m*/*z* 125.02 (C_6_H_5_O_3_) is detected, we can deduce that the newly added hydroxyl group is linked to the A ring; (2) If the fragment ions at *m*/*z* 151.04 and *m*/*z* 136.02 are observed at the same time, we can confirm that the newly added hydroxyl group is attached to the D ring of medicarpin and the ring cleavage is at the bond of C11-C11a.

#### 2.2.8. Analysis of Metabolites (**M139**–**M149**) Having the Skeleton of Demethylated, Hydrogenated and Hydroxylated Medicarpin

**M140**–**M146** showed [M − H]^−^ at *m*/*z* 353.03, thus their molecular formulae were identified as C_15_H_14_O_8_S. In their MS^2^ spectra, *m*/*z* 273.08 (C_15_H_13_O_5_) generated by a neutral loss 79.95 Da (SO_3_) was detected. Hence, **M140**–**M146** were demethylated, hydrogenated and hydroxylated medicarpin sulfates.

**Figure 12:**
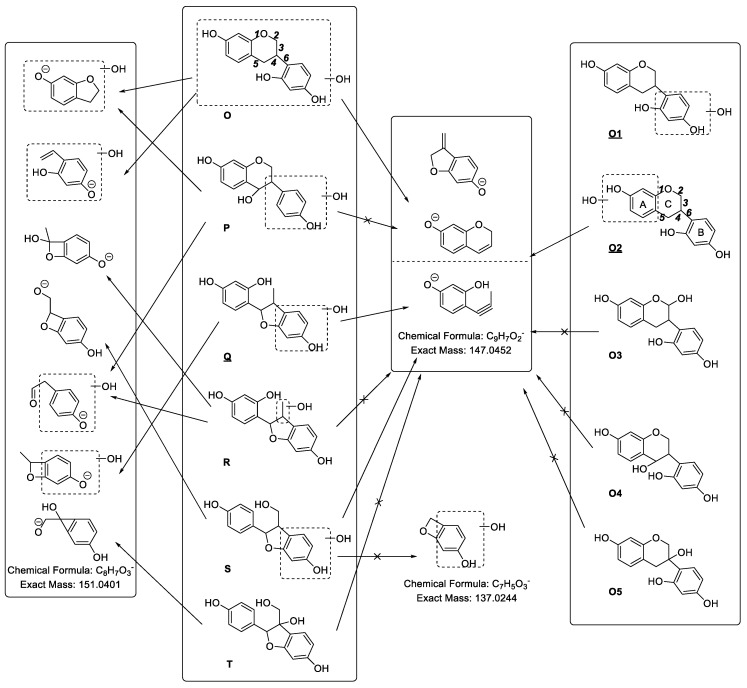
The probable structures (**O1**, **O2**, **Q**) of the aglycon of **M144**.

**M144** yielded a series of characteristic fragment ions at *m*/*z* 273.0782 ([Aglycon − H]^−^), *m*/*z* 163.0437 (C_9_H_7_O_3_), *m*/*z* 161.0315 (C_9_H_5_O_3_), *m*/*z* 151.0428 (C_8_H_7_O_3_), *m*/*z* 147.0451 (C_9_H_7_O_2_), *m*/*z* 137.0308 (C_7_H_5_O_3_), and *m*/*z* 121.0276 (C_7_H_5_O_2_). Based on the fragment ion at *m*/*z* 151.0428 (C_8_H_7_O_3_), we could deduce that the aglycon of **M144** might be **O**, **P**, **Q**, **R**, **S** or **T**. According to the fragment ion at *m*/*z* 147.0451 (C_9_H_7_O_2_), we could exclude **P**, **R** and **T**. Besides, based on the fragment ion at *m*/*z* 137.0308 (C_7_H_5_O_3_), we could exclude **E**. Therefore, we could determine that the aglycon of **M144** was **O** or **Q**. Furthermore, based on the fragment ion at *m*/*z* 147.0451 (C_9_H_7_O_2_), we finally deduced that the aglycon of **M144** was **O1**, **O2** or **Q** (Figure 12). The MS^2^ spectrum of **M144** is shown in Figure S51.

The molecular formulae of **M147**–**M149** were predicted as C_21_H_22_O_11_ in line with [M − H]^−^ at *m*/*z* 449.11. The fragment ion at *m*/*z* 273.08 (C_15_H_13_O_5_) formed by losing 176.03 Da (C_6_H_8_O_6_) from [M − H]^−^ was detected in the MS^2^ spectra. Hence, **M147**–**M149** were demethylated, hydrogenated and hydroxylated medicarpin glucuronides.

#### 2.2.9. Analysis of Metabolites (**M150**–**M156**) Having the Skeleton of Demethylated and Dihydroxylated Medicarpin

**M150**–**M151** showed [M − H]^−^ at *m*/*z* 463.09 and the molecular formulae were calculated to be C_21_H_20_O_10_. **M152**–**M156** showed [M − H]^−^ at *m*/*z* 367.01, indicating the molecular formulae of C_15_H_12_O_9_S. In their MS^2^ spectra, the fragment ion at *m*/*z* 287.06 (C_15_H_11_O_6_) was detected. Compared with C_16_H_14_O_4_ of medicarpin, it had a less CH_2_ and two more oxygen atoms. As a result, medicarpin might undergo demethylation and dihydroxylation. Therefore, **M150**–**M151** were glucuronides of demethylated and dehydroxylated medicarpin, while **M152**–**M156** were sulfates of demethylated and dehydroxylated medicarpin.

#### 2.2.10. Analysis of Metabolites (**M157**–**M161**) Having the Skeleton of Dihydroxylated Medicarpin

**Figure 13:**
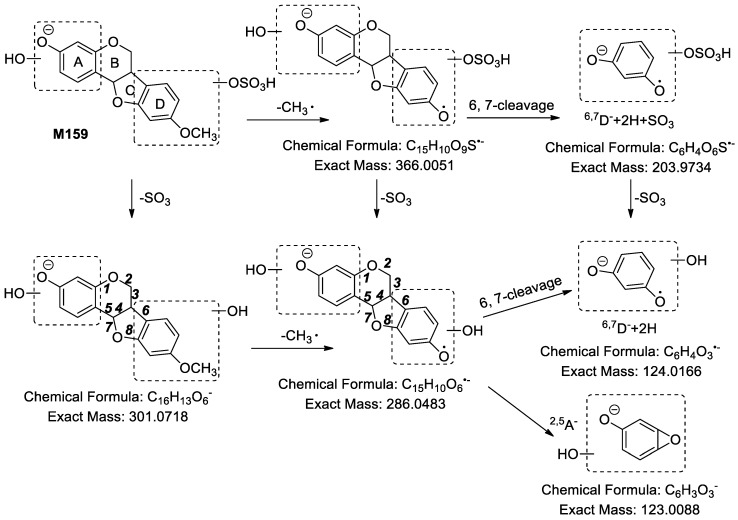
The proposed fragmentation pathways of **M159**.

**M157**–**M159** showed [M − H]^−^ at *m*/*z* 381.03 and their molecular formulae were determined as C_16_H_14_O_9_S. In MS^2^ spectra, *m*/*z* 381.03 produced [Aglycon − H]^−^ at *m*/*z* 301.07 (C_16_H_13_O_6_) by the loss of 79.95 Da (SO_3_). The elemental composition difference between medicarpin (C_16_H_14_O_4_) and the aglycon of **M157**–**M159** was O_2_. Therefore, **M157**–**M159** were identified as sulfates of dihydroxylated medicarpin. In MS^2^ spectra of **M159**, fragment ions at *m*/*z* 366.0054 (C_15_H_10_O_9_S, [M − H]^−^ − CH_3_•), *m*/*z* 301.0711 ([Aglycon − H]^−^), *m*/*z* 286.0477 ([Aglycon − H]^−^ − CH_3_•) and *m*/*z* 203.9750 (C_6_H_4_O_6_S, ^6,7^D^−^ + 2H + SO_3_) were detected. Subsequently, *m*/*z* 286.0477 yielded a series of fragment ions at *m*/*z* 162.0347 (C_9_H_6_O_3_, ^2,5^D^−^ − CH_3_•), *m*/*z* 161.0269 (C_9_H_5_O_3_, ^6,7^A^−^ − 2H), *m*/*z* 149.0301 (C_8_H_5_O_3_, ^3,4,7^A^−^ − H), *m*/*z* 137.0229 (C_7_H_5_O_3_, ^3,5^A^−^), *m*/*z* 133.0328 (C_8_H_5_O_2_, ^6,7^A^−^ − 2H − CO), *m*/*z* 124.0254 (C_6_H_4_O_3_, ^6,7^D^−^ + 2H − CH_3_•) and *m*/*z* 123.0137 (C_6_H_3_O_3_, ^2,5^A^−^). Based on the fragment ion at *m*/*z* 123.0137 (C_6_H_3_O_3_, ^2,5^A^−^), we assured that one hydroxyl group must be linked to the A ring. Besides, the fragment ion at *m*/*z* 124.0254 (C_6_H_4_O_3_, ^6,7^D^−^ + 2H − CH_3_•) suggested that one hydroxyl group must be linked to the D ring. Furthermore, according to the fragment ion at *m*/*z* 203.9750 (C_6_H_4_O_6_S), we could determine that the sulfonic group was linked to the hydroxyl group of D ring. The fragmentation pathways of **M159** are shown in Figure 13. The MS spectrum of **M159** is shown in Figure S52.

#### 2.2.11. Analysis of Metabolites M162 Having the Skeleton of Hydrogenated and Dihydroxylated Medicarpin

Its molecular formula was calculated to be C_16_H_16_O_6_ based on [M − H]^−^ at *m*/*z* 303.0861. Compared with the C_16_H_14_O_4_ of medicarpin, it had two more hydrogen atoms and two more oxygen atoms. Accordingly, **M162** was determined as hydrogenated and dihydroxylated medicarpin.

#### 2.2.12. Analysis of Metabolites **M163**–**M164** Having the Skeleton of Hydrogenated and Trihydroxylated Medicarpin

**M163** showed [M − H]^−^ at *m*/*z* 399.0528 and its molecular formula was predicted to be C_16_H_16_O_10_S. In its MS^2^ spectra, fragment ions at *m*/*z* 381.0296 (C_16_H_13_O_9_S), *m*/*z* 301.0664 (C_16_H_13_O_6_) and *m*/*z* 286.0504 (C_15_H_10_O_6_) were detected. Therefore, **M163** was regarded as hydrogenated and trihydroxylated medicarpin sulfate. The molecular formula of **M164** was calculated to be C_17_H_18_O_10_S based on [M − H]^−^ at *m*/*z* 413.0521. In its MS^2^ spectra, fragment ions at *m*/*z* 381.0275 (C_16_H_13_O_9_S), *m*/*z* 301.0671 (C_16_H_13_O_6_) and *m*/*z* 286.0517 (C_15_H_10_O_6_) were detected. Therefore, **M164** was determined as hydrogenated, trihydroxylated and methylated medicarpin sulfate.

#### 2.2.13. Analysis of Metabolite **M165** Having the Skeleton of Demethylated, Hydrogenated and Dihydroxylated Medicarpin

**M165** showed [M − H]^−^ at *m*/*z* 369.0297 and its molecular formula was predicted to be C_15_H_14_O_9_S. In its MS^2^ spectra, fragment ions at *m*/*z* 351.0135 (C_15_H_1__1_O_8_S) and *m*/*z* 271.0607 (C_15_H_1__1_O_5_) were detected, indicating two sequential neutral losses of 18.01 Da (H_2_O) and 79.95 Da (SO_3_). Compared with C_16_H_1__3_O_4_ of medicarpin, *m*/*z* 271.0607 had a less CH_2_ and one more oxygen atom. Therefore, **M165** was determined as a demethylated, hydrogenated and dihydroxylated medicarpin sulfate.

### 2.3. Distribution of 165 Metabolites in Rats

**Figure 14:**
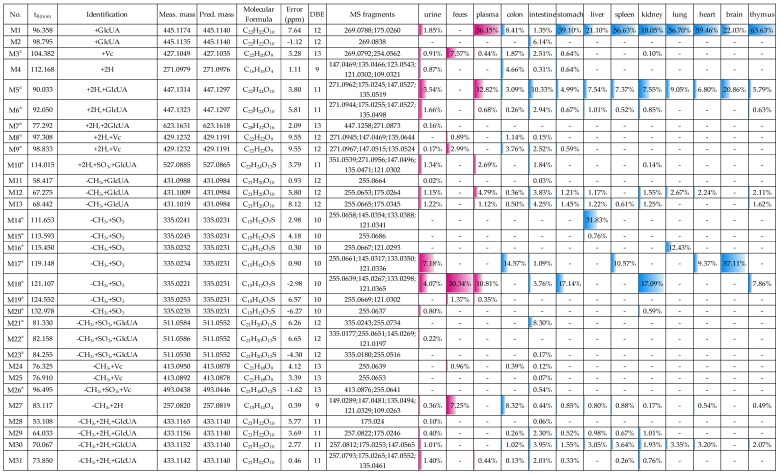

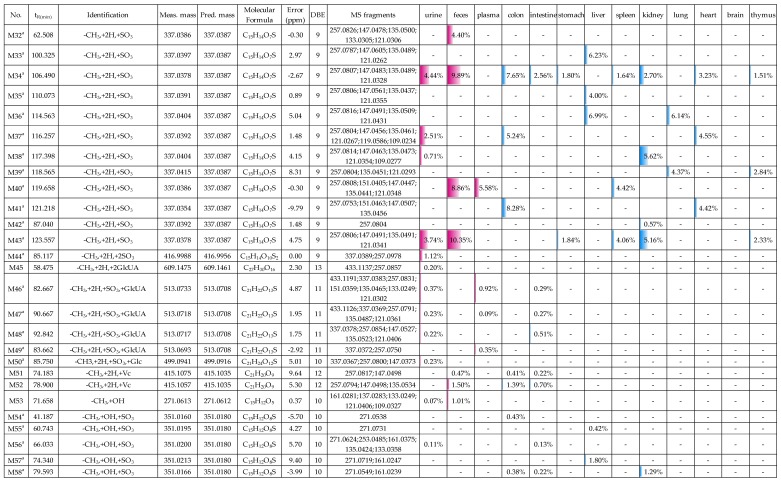

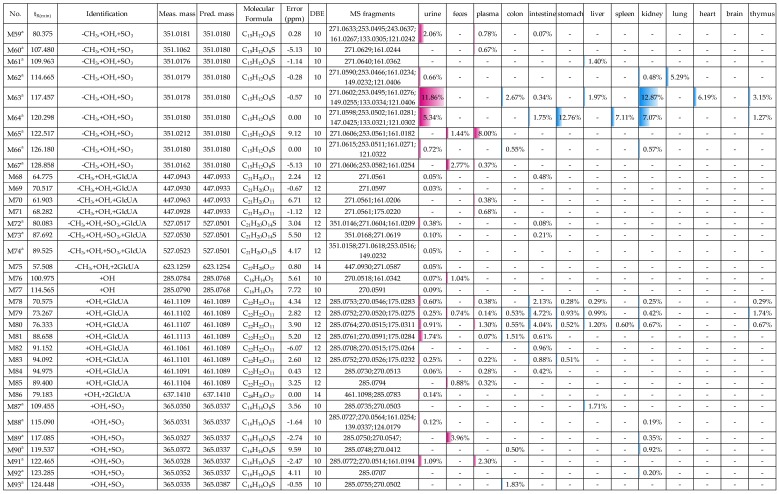

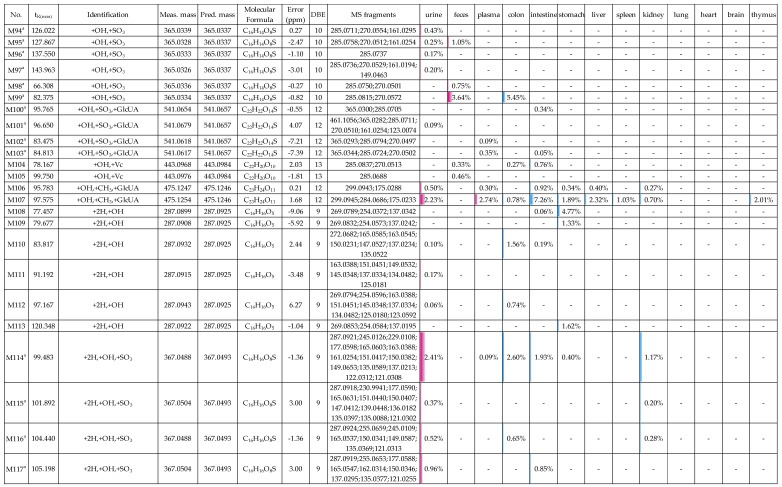

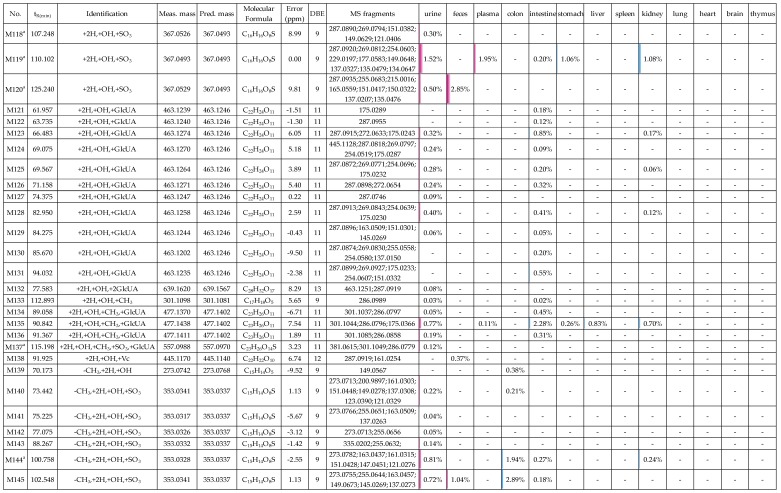

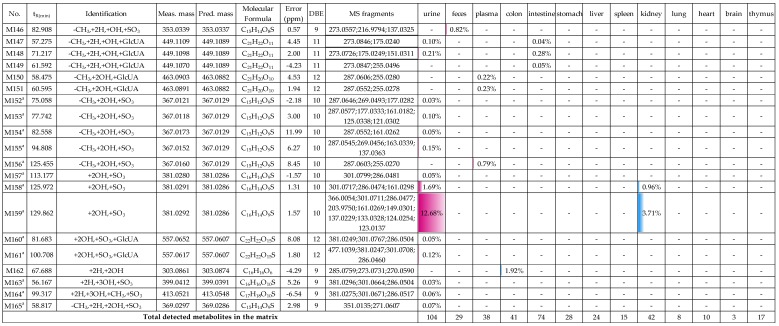
The distribution of 165 metabolites of medicarpin and their relative contents in 13 biosamples (^a^, new compounds). The relative content of a metabolite in each biosample was calculated by (peak area of a metabolite/total peak area of all detected metabolites) × 100%.

The distribution of 165 metabolites (including 104 metabolites in urine, 29 metabolites in feces, 38 metabolites in plasma, 41 metabolites in the colon, 74 metabolites in the intestine, 28 metabolites in the stomach, 24 metabolites in the liver, 15 metabolites in the spleen, 42 metabolites in the kidney, 8 metabolites in the lung, 10 metabolites in the heart, 3 metabolites in the brain and 17 metabolites in the thymus) of medicarpin and their relative contents in each biological sample are shown in Figure 14. The relative content of a metabolite in each biosample was calculated by (peak area of a metabolite in the sample/total peak area of all metabolites detected in the sample) × 100%. The peak area of a metabolite was calculated from its extracted ion chromatogram.

From Figure 14, we could find that: (1) in urine, **M159** (12.68%, dihydroxylated medicarpin sulfate), **M63** (11.86%, demethylated and hydroxylated medicarpin sulfate), **M17** (7.18%, demethylated medicarpin sulfate), and **M64** (5.34%) were four major metabolites and all of them were sulfates; it is an interesting phenomenon that sulfates other than glucuronides are major metabolites in urine, and the cause is unclear; (2) in feces, **M18** (20.34%), **M43** (10.35%), **M34** (9.89%), **M40** (8.86%), **M3** (7.57%, Vc conjugate), and **M27** (7.25%) were six major metabolites with a relative content higher than 5%, and three of them (**M43**, **M34**, **M40**) were demethylated and hydrogenated medicarpin sulfates; (3) in plasma, **M1** (36.15%, medicarpin glucuronide), **M5** (12.82%, vestitol-1’-*O*-glucuronide), **M18** (10.81%), **M65** (8.00%),and **M40** (5.58%) were major metabolites; (4) **M1** was the most abundant metabolite in the stomach, liver, spleen, kidney, lung, heart, and thymus. **M5**, **M14** (demethylated medicarpin) were the most abundant metabolites in intestine and liver, respectively. **M17** (demethylated medicarpin sulfate) was the most abundant metabolite in the colon and brain; (5) **M1**, **M5** were detected in all 10 organs; **M30** (demethylated and hydrogenated medicarpin glucuronide) was detected in nine organs (except the brain); **M12** and **M27** were detected in eight organs; therefore, these five metabolites were widely distributed in vivo. Considering the high abundance and wide distribution, we suggest that the bioactivities of these metabolites deserve further research in order to fully explain the pharmacological action mechanism of medicarpin. In addition, the reason for specific distribution of some metabolites (e.g., **M2**, **M14**, **M20**, **M35**, **M40**, **M54**, **M109**) also need further investigation.

The metabolic reactions of medicarpin included demethylation, hydrogenation, hydroxylation, glucuronidation, sulfation, glycosylation, methylation, and conjunction of vitamin C. The relative contents of metabolic reactions and phase I metabolites in each biosample are shown in Table 1, which were calculated by summing the relative contents of all metabolites generated through it and all phase I metabolites, respectively. In urine, the contents of sulfation metabolites, glucuronidation metabolites and vitamin C conjugates were 74.60%, 25.83% and 1.08%, respectively. In feces, the contents of sulfation metabolites, glucuronidation metabolites and vitamin C conjugates were 73.53%, 1.62% and 15.54%, respectively. This indicated that medicarpin was mainly excreted in the form of sulfates. In plasma, the major metabolic reaction was glucuronidation (relative content: 67.87%). The glycosylation metabolite (**M50**) was only found in urine. Furthermore, the contents of phase I metabolites of medicarpin in most biosamples were very low (<10%), except in the colon (17.57%), which suggested that phase II metabolites are major metabolites. 

## 3. Materials and Methods

### 3.1. Chemicals and Reagents

Medicarpin (Molecular formula: C_16_H_14_O_4_, exact mass: 270.0892 Da) was isolated from the Maackia amurensis Radix by chromatographic methods, including 200–300 mesh normal phase silica gel (Sinopharm Chemical Reagent Co., Ltd., Shanghai, China) column chromatography and reversed phase C18 silica gel (YMC, YMC Co., Ltd., Kyoto, Japan) column chromatography. The optical rotation angle was ${\left[\alpha \right]}_{25}^{L}$ = −19.92 (c = 5, CH_3_OH). Hence, its structure was determined to be (−)-3-hydroxy-9-methoxypterocarpan or (−)-medicarpin on the basis of UV, NMR, and MS data. Its purity was greater than 98% by the peak area normalization method using HPLC-UV analysis at 280 nm. Formic acid (Fisher scientific, Fair lawn, NJ, USA), acetonitrile (Fisher scientific), and methanol (Tianjin Damao Chemicals, Tianjin, China) were of HPLC grade. Ultrapure water was prepared using a Milli-Q water purification system (Millipore, Billerica, MA, USA). Analytical grade sodium carboxymethyl cellulose (CMC-Na) was purchased from Tianjin Guangfu Fine Chemical Research Institute (Tianjin, China). All other chemicals and reagents were of analytical grade.

### 3.2. Animals and Drug Administration

Eight male Sprague-Dawley rats (220–250 g) were obtained from the Experimental center of Peking University Health Science Center (Beijing, China). All rats were housed in a controlled animal room. Before oral gavage, the rats were randomly divided into two groups (a drug group and a blank group, four rats per group). Each rat was housed in a metabolic cage with water and food ad libitum for three days. Medicarpin was suspended in 0.5% CMC-Na solution and administrated at a dose of 100 mg/kg (body weight) to the drug group rats once a day for four days, while the blank group were given the same volume of 0.5% CMC-Na. All animal treatments were approved by the Biomedical Ethical Committee of Peking University (approval No. LA2015134).

### 3.3. Sample Collection and Preparation

Urine and feces samples were collected for the first three days after drug administration; plasma samples and organ samples were collected on the last day one hour after drug administration.

Urine samples: All urine samples from the same group were merged and evaporated to dryness at 40 °C using a Laborota 4001 rotator evaporator (Heidolph Instruments GmbH & Co., Schwabach, Germany) under vacuum, respectively. Then, each group added 10 volumes of methanol (10 mL methanol/g residue) and extracted ultrasonically for 30 min. The extracts were filtered and evaporated to dryness at 40 °C. Finally, the residues were dissolved in 2 volumes of methanol (2 mL methanol/g residue), filtered through 0.22 μm membranes, and stored at −80 °C before further analysis.

Feces samples: All feces samples from the same group were dried at 50 °C and crushed into powder, respectively. Then, each group was ultrasonically extracted with 10 volumes (mL/g) of methanol for 30 min and filtered, after that the filtrate was evaporated to dryness at 40 °C. The residues were dissolved in 4 volumes of methanol (4 mL methanol/g residue), filtered through 0.22 μm membranes before being stored at −80 °C.

Plasma samples: An hour after the last drug administration, the blood was collected in heparin tubes by cardiac puncture technique in groups through anesthetizing with an intraperitoneal injection of chloral hydrate. Plasma was obtained after centrifugation at 2292× *g* (5000 rpm), 4 °C for 15 min using a 3–30 K refrigerated centrifuge (Sigma Laborzentrifugen GmbH, Osterode am Harz Germany). Afterwards, each plasma sample was ultrasonically extracted with 10 volumes (mL/mL) of methanol for 30 min in order to remove the protein content and then centrifuged for 15 min at 2292× *g* (5000 rpm), 4 °C. The supernatant of each group was evaporated to dryness under vacuum by a rotator evaporator. Then the residue was dissolved in 1 volume of methanol (1 mL methanol/g residue) and filtered through 0.22 μm membranes before stored at −80 °C.

Organ samples: After collecting blood via cardiac puncture, the heart, liver, spleen, lungs, kidneys, colon, intestine, stomach, brain, thymus were rapidly removed and washed three times with cold saline to flush away the surface blood and other residual substances. Later, they were preserved at −80 °C after drying with filter papers. Then, each kind of the organs from the same group were combined and weighed, as well as added 5 times volumes of methanol (5 mL methanol/g tissue). The mixture was homogenized by a T10 homogenizer (IKA Co., Ltd., Staufen, Germany) under low temperature conditions, and ultrasonically extracted by a KQ-2200B ultrasonic cleaning machine (Kunshan Ultrasonic Instruments Co., Ltd., Jiangsu, China) for an hour and then centrifuged at 2292× *g* (5000 rpm) at 4 °C for 15 min. Additionally, the supernatant was concentrated and dried in a vacuum at 40 °C, reconstituted with 10 times volume of methanol, and maintained at −80 °C. And, before LC-MS analysis they shall be filtered through 0.22 μm membranes.

### 3.4. Instrumentation and Analytical Conditions

The chromatographic separation was performed on the Agilent HPLC instrument using a Phenomenex Gemini C18 column (250 mm × 4.60 mm, 5 μm). The column oven temperature was maintained at 35 °C and the volume injected was 10 μL. The mobile phases were water (contained 0.1% formic acid, *v*/*v*) (A) and acetonitrile (B). The gradient elution program was as follows: 0–10 min, 3% B; 10–130 min, 3–40% B; 130–140 min, 40–100% B; 140–155 min, 100% B. The flow rate was 1.0000 mL/min.

HPLC-ESI-IT-TOF-MS^n^ analysis was performed on a Shimadzu LC-MS-IT-TOF instrument, which consists of a CBM-20A system controller, two LC-20AD pumps, an SIL-20AC autosampler, a CTO-20A column oven, an SPD-M20A PDA detector, an ESI ion source, and an IT-TOF mass spectrometer using the same separation conditions. The mass spectrometer flow rate was 0.2000 mL/min that was split from HPLC effluent and the detection mode included positive ion (ESI+) and negative ion (ESI−) mode with a full-scan covering *m*/*z* 100–1000 (MS) and *m*/*z* 50–1000 (MS^2^ and MS^3^). The other optimal conditions were set as follows: heat block and curved desolvation line temperature was 250 °C and nebulizing nitrogen gas flow was 1.5 mL/min; the interface voltage was (+)-4.5 kV and (−)-5.5 kV and the detector voltage was 1.7 kV. In addition, the ion accumulation time was 30 ms and the relative collision-induced dissociation energy was 70%. The mass range *m*/*z* 50–3000 was calibrated by a trifluoroacetic acid sodium solution (2.5 mM). All data were acquired and analyzed by Shimadzu LCMS solution Version 3.60, Formula Predictor Version 1.2, and Accurate Mass Calculator (Shimadzu, Kyoto, Japan).

## 4. Conclusions

In this paper, the metabolites of medicarpin and their distributions in rats were systematically studied for the first time, and 165 new metabolites (13 phase I metabolites and 152 phase II metabolites) were tentatively identified by HPLC-ESI-IT-TOF-MS^n^, including 104 metabolites in urine, 29 in feces, 38 in plasma, 41 in the colon, 74 in the intestine, 28 in the stomach, 24 in the liver, 15 in the spleen, 42 in the kidney, eight in the lung, 10 in the heart, three in the brain and 17 in the thymus. Eighty-six sulfate metabolites, five Vitamin C conjugates (**M3**, **M8**, **M9**, **M24**, **M25**) and two hydroxylated medicarpin glucuronides (**M5** and **M6**) were regarded as potential new compounds by retrieving information from the Scifinder database. Besides, based on the structure of these metabolites, the metabolic pathways of medicarpin were proposed. The metabolic reactions of medicarpin included demethylation, hydrogenation, hydroxylation, glucuronidation, sulfation, glycosylation, methylation and conjunction of vitamin C, among which sulfation and glucuronidation were the major phase II metabolic reactions. Five metabolites (**M1**, **M5**, **M30**, **M12**, **M27**) were widely distributed to nine or ten organs, and the specific distribution of lots of metabolites (e.g., **M2**, **M14**, **M20**, **M35**, **M40**, **M54**, **M109**) were also discovered. Furthermore, we found that isomerization of medicarpin must occur in vivo. This study is the first comprehensive report on the metabolism of pterocarpans in animals. The results will facilitate the understanding of the metabolism of medicarpin, and will provide a scientific basis for further pharmacological studies on medicarpin and for metabolism research of other pterocarpans in animals.

## Figures and Tables

**Table 1:** Relative contents of metabolic reactions and phase I metabolites of medicarpin in 13 biological samples (%).

Biosample	Relative Contents (%)								
	Phase I Reaction			Phase II Reaction					Phase I Metabolites
	-CH_3_	+2H	+OH	+SO_3_	+GlcUA	+Vc	+Glc	+CH_3_	
urine	55.14	37.01	57.79	74.60	25.83	1.08	0.23	3.93	1.78
feces	71.51	44.43	21.55	73.53	1.62	15.54	—	— ^1^	9.30
plasma	36.58	25.72	22.78	36.17	67.87	0.44	—	3.16	—
colon	57.95	58.48	28.30	55.81	16.61	9.23	—	0.78	17.57
intestine	39.56	41.46	36.17	15.62	78.04	7.60	—	11.23	0.99
stomach	39.46	22.38	26.67	35.00	54.57	1.23	—	2.50	9.20
liver	62.62	31.43	13.33	57.11	42.09	—	—	3.55	0.80
spleen	33.84	23.45	8.74	27.79	71.33	—	—	1.03	0.88
kidney	60.90	30.46	34.93	63.43	36.30	0.10	—	1.67	0.17
lung	34.25	22.91	5.29	28.22	71.78	—	—	—	—
heart	33.74	22.74	6.19	27.76	71.70	—	—	—	0.54
brain	57.11	20.86	—	57.11	42.89	—	—	—	—
thymus	25.25	15.66	9.12	18.96	80.54	—	—	2.01	0.49

^1^ —, undetected.

## Supplementary Materials

Figures S1–S52 are available online.
