# Supplementary material for: Metabolites of Medicarpin and Their Distributions in Rats
Source: Molecules. 2019 May 22;24(10):1966. doi: 10.3390/molecules24101966 (PMC6572127; doi:10.3390/molecules24101966)
Supplement: Supplementary file 1 [file molecules-24-01966-s001.pdf]

Supplementary materials

Metabolites of Medicarpin and Their Distributions in Rats

Hong-Yan Wang [1], Teng Li [1], Rui Ji [1,2], Feng Xu [1,*], Guang-Xue Liu [1], Yao-Li Li [1], Ming-Ying Shang [1,*] and Shao-Qing Cai [1]

[1] State Key Laboratory of Natural and Biomimetic Drugs, School of Pharmaceutical Sciences, Peking University, No. 38, Xueyuan Road, Beijing 100191, China; wanghongyan@bjmu.edu.cn (H.-Y.W.); tengli1175@163.com (T.L.); jiruiwonder@163.com (R.J.); guangxl@bjmu.edu.cn (G.-X.L.); liyaoli123456@163.com (Y.-L.L.); sqcai@bjmu.edu.cn (S.-Q.C.)

[2] School of Pharmacy, Heilongjiang University of Chinese Medicine, No.24, Heping Road, Xiangfang District, Harbin 150040, China

* Correspondence: xufeng76@bjmu.edu.cn (F.X.); myshang@bjmu.edu.cn (M.-Y.S.); Tel.: +86-10-8280-2534 (F.X. & M.-Y.S.)

1. The EICs of 165 metabolites in rats (Figure S1–S44)

(1) metabolites (**M1–M3**) having the skeleton of medicarpin

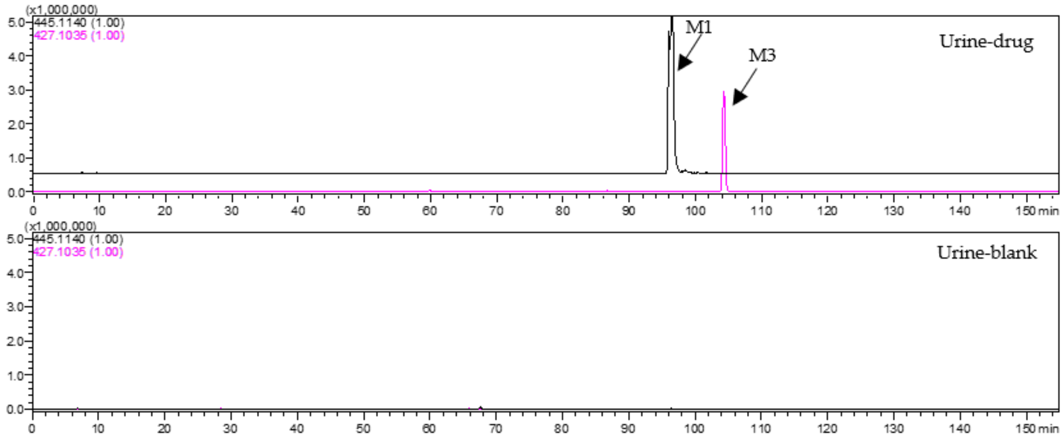

Figure S1. EICs of *m*/*z* 445.1140 and *m*/*z* 427.1035 in drug urine and blank urine in ESI− mode.

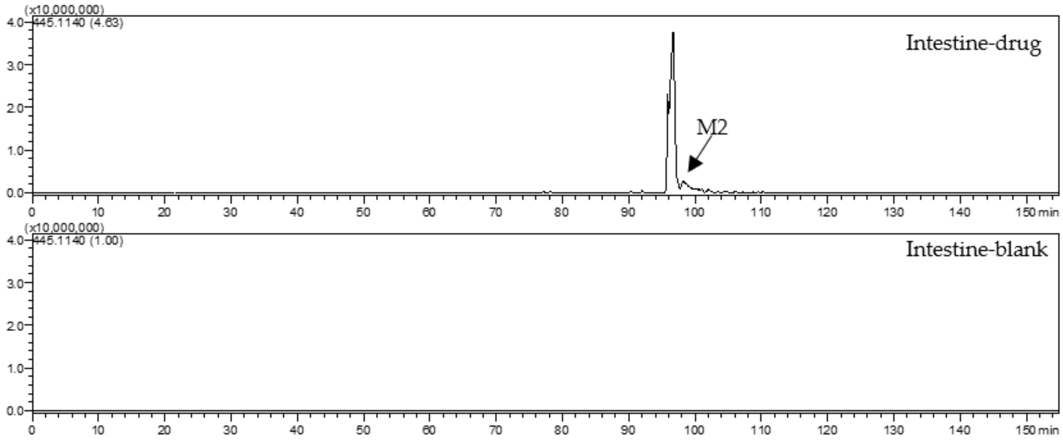

Figure S2. EICs of *m*/*z* 445.1140 in drug intestine and blank intestine in ESI− mode.

(2) metabolites (**M4-M10**) having the skeleton of hydrogenated medicarpin

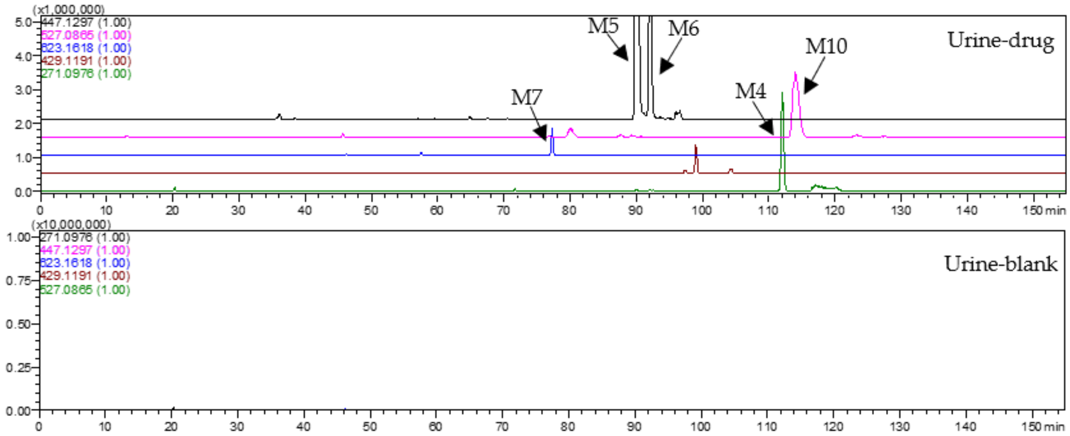

Figure S3. EICs of *m*/*z* 447.1297, *m*/*z* 527.0865, *m*/*z* 623.1618, *m*/*z* 429.1191 and *m*/*z* 271.0976 in drug urine and blank urine in ESI− mode.

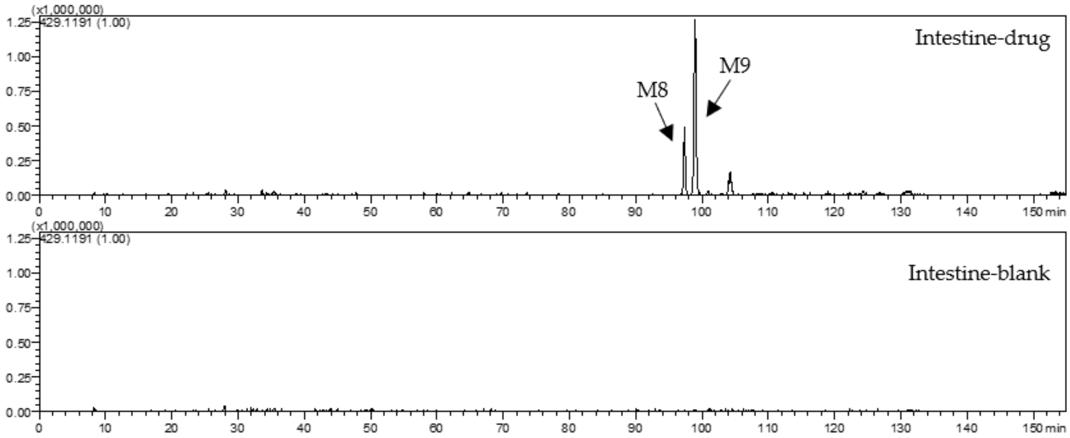

Figure S4. EICs of *m*/*z* 429.1191 in drug intestine and blank intestine in ESI− mode.

(3) metabolites (**M11–M26**) having the skeleton of demethylated medicarpin

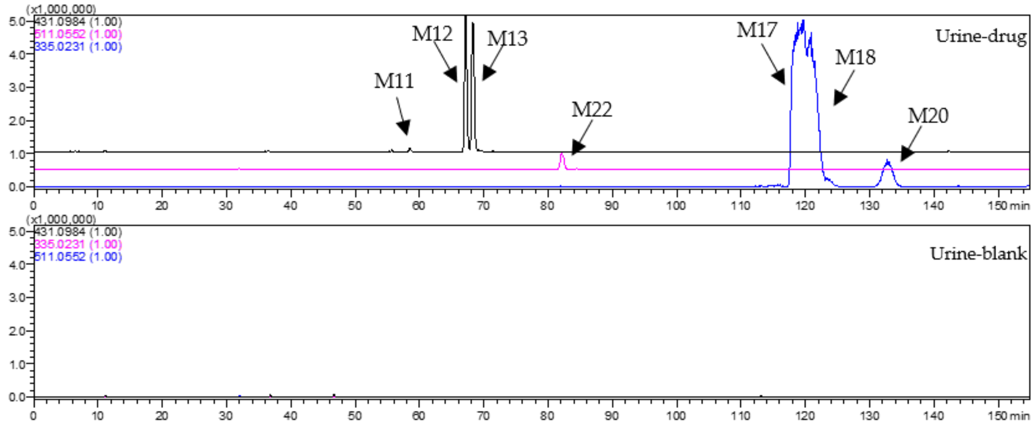

Figure S5. EICs of *m*/*z* 431.0984, *m*/*z* 511.0552 and *m*/*z* 335.0231 in drug urine and blank urine in ESI− mode.

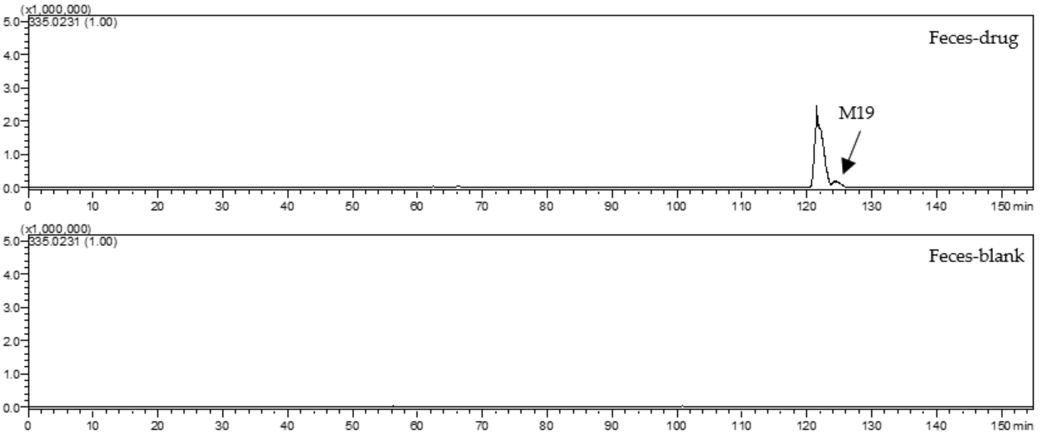

Figure S6. EICs of *m/z* 335.0231 in drug feces and blank feces in ESI− mode.

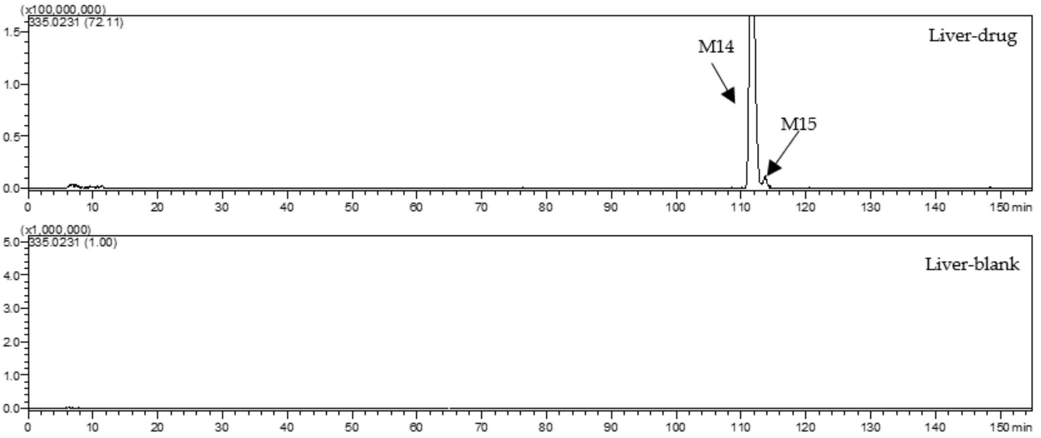

Figure S7. EICs of *m/z* 335.0231 in drug liver and blank liver in ESI− mode.

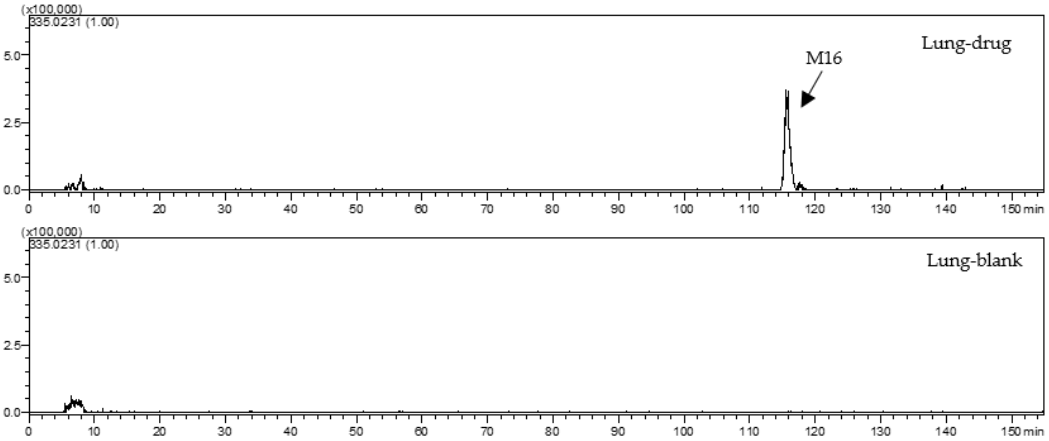

Figure S8. EICs of *m/z* 335.0231 in drug lung and blank lung in ESI− mode.

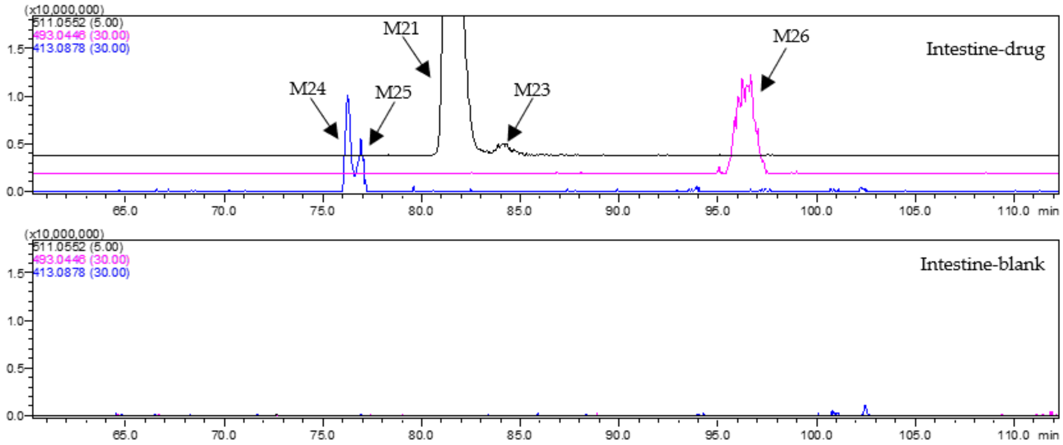

Figure S9. EICs of *m/z* 511.0552, *m/z* 493.0446 and *m/z* 413.0873 in drug intestine and blank intestine in ESI− mode.

(4) metabolites (**M27–M52**) having the skeleton of demethylated and hydrogenated medicarpin

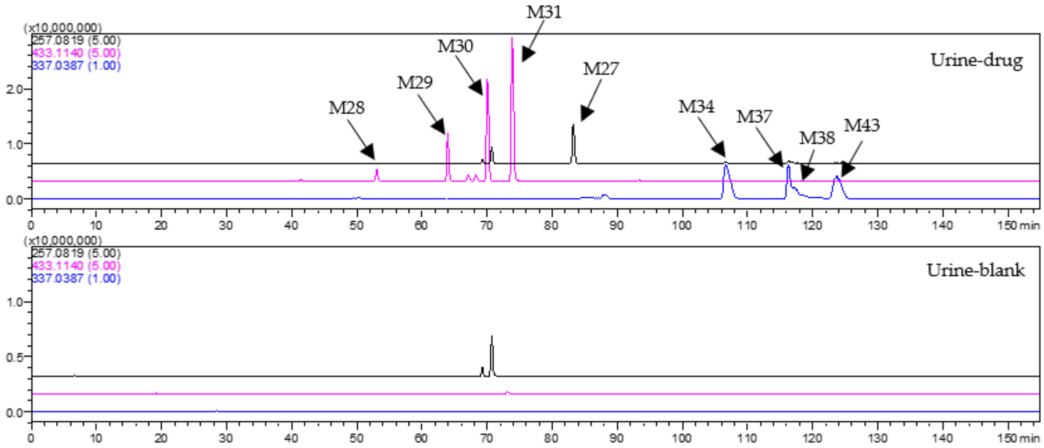

Figure S10. EICs of *m/z* 257.0819, *m/z* 433.1140 and *m/z* 337.0387 in drug urine and blank urine in ESI− mode.

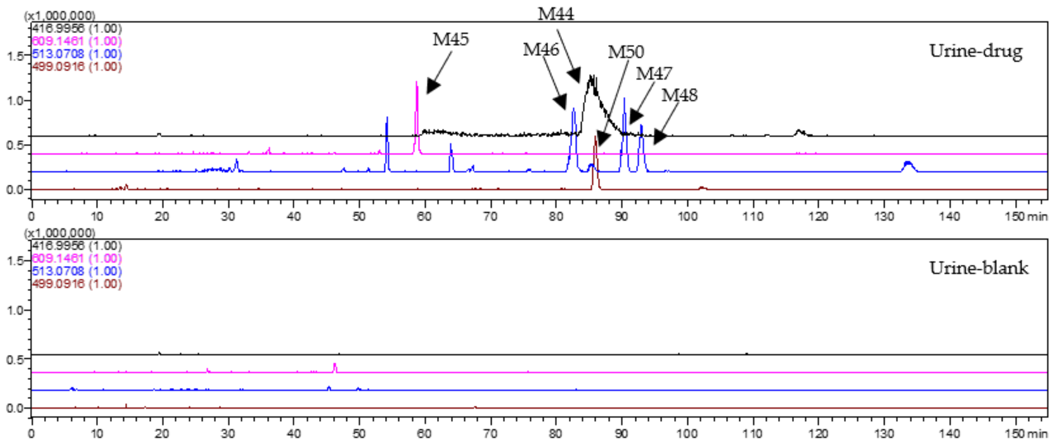

Figure S11. EICs of *m/z* 416.9956, *m/z* 609.1461, *m/z* 513.0708 and *m/z* 499.0916 in drug urine and blank urine in ESI− mode.

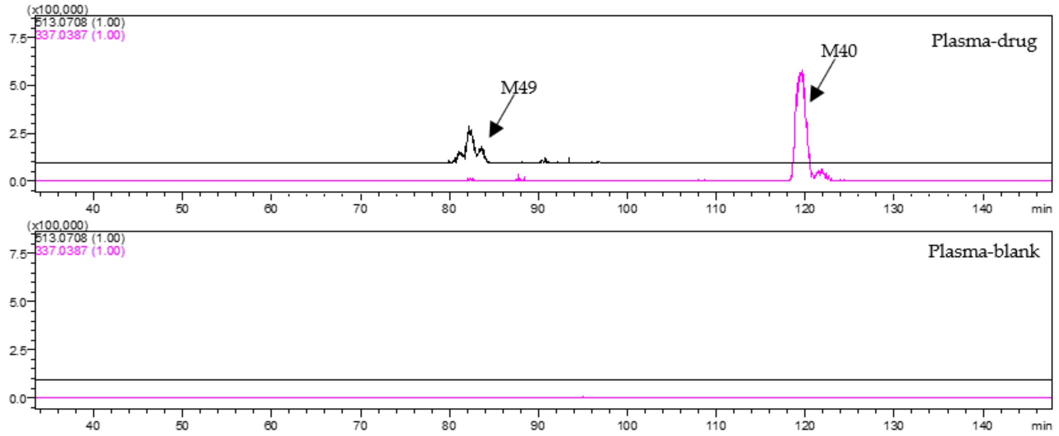

Figure S12. EICs of *m/z* 513.0708 and *m/z* 337.0387 in drug plasma and blank plasma in ESI− mode.

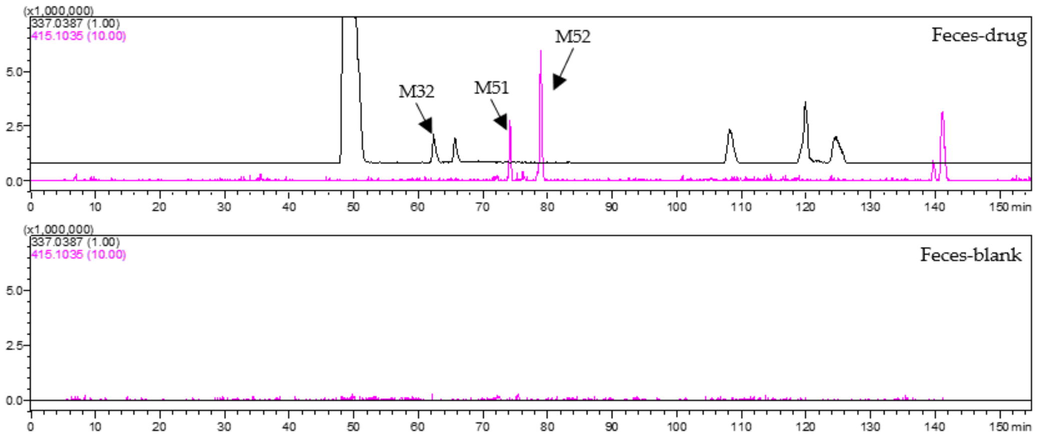

Figure S13. EICs of *m/z* 337.0387 and *m/z* 415.1035 in drug feces and blank feces in ESI− mode.

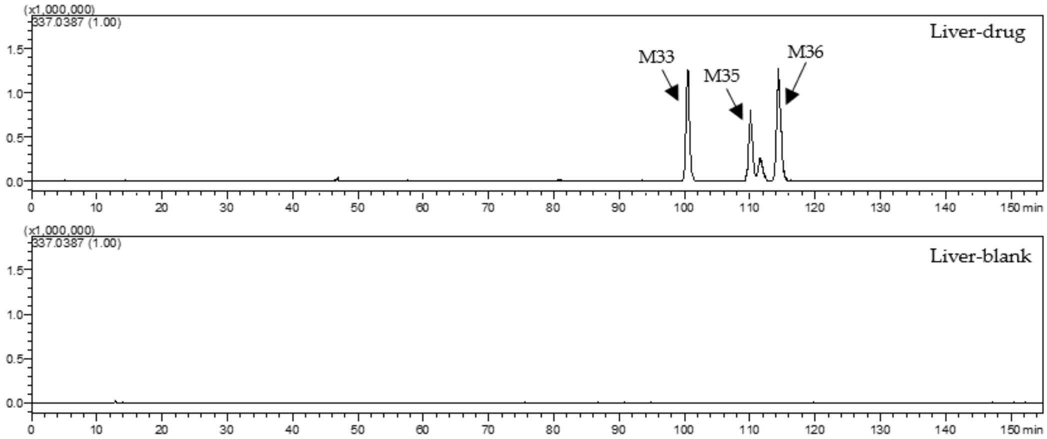

Figure S14. EICs of *m/z* 337.0387 in drug liver and blank liver in ESI− mode.

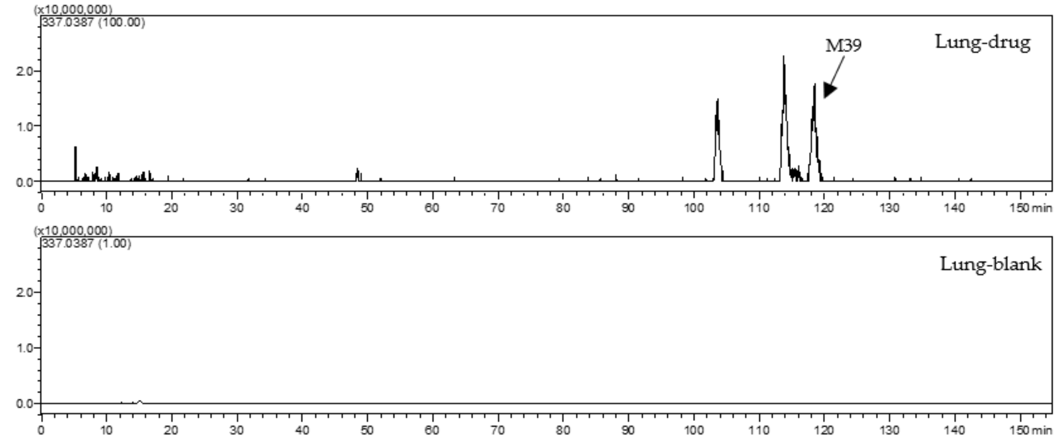

Figure S15. EICs of m/z 337.0387 in drug lung and blank lung in ESI− mode.

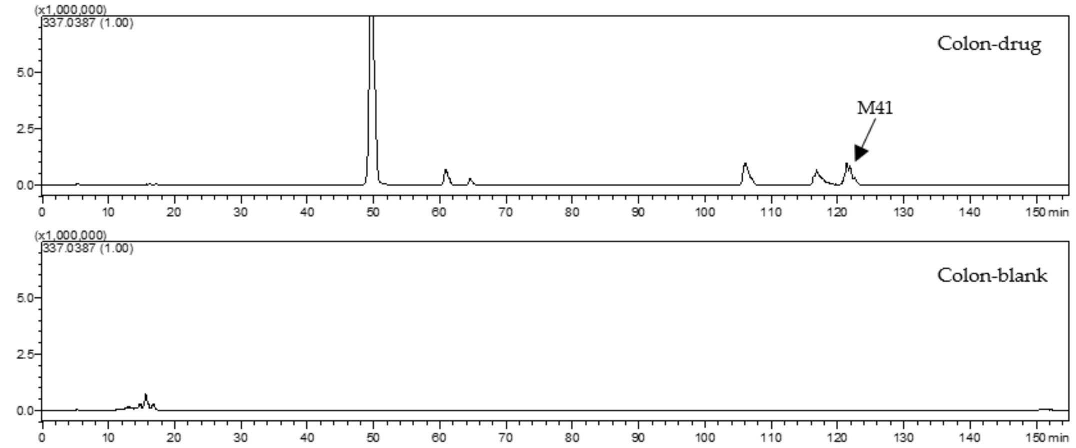

Figure S16. EICs of m/z 337.0387 in drug colon and blank colon in ESI− mode.

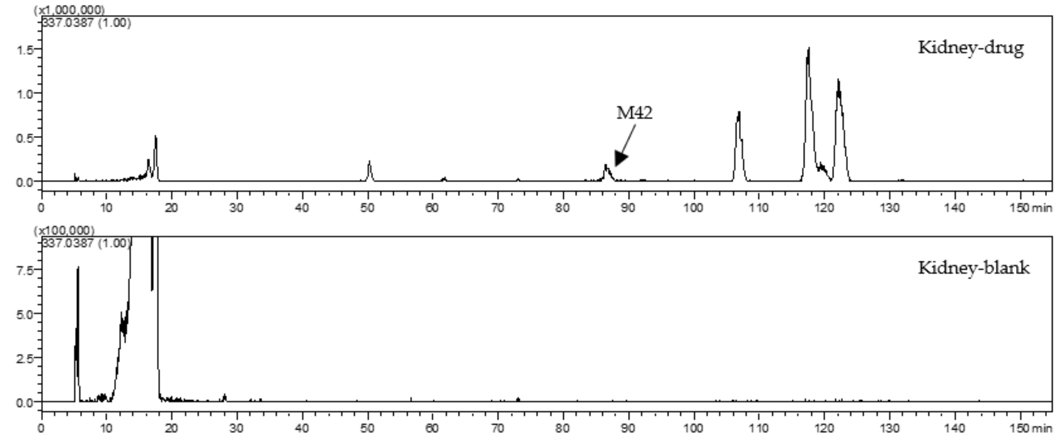

Figure S17. EICs of m/z 337.0387 in drug kidney and blank kidney in ESI− mode.

(5) metabolites (**M53–M75**) having the skeleton of demethylated and hydroxylated medicarpin

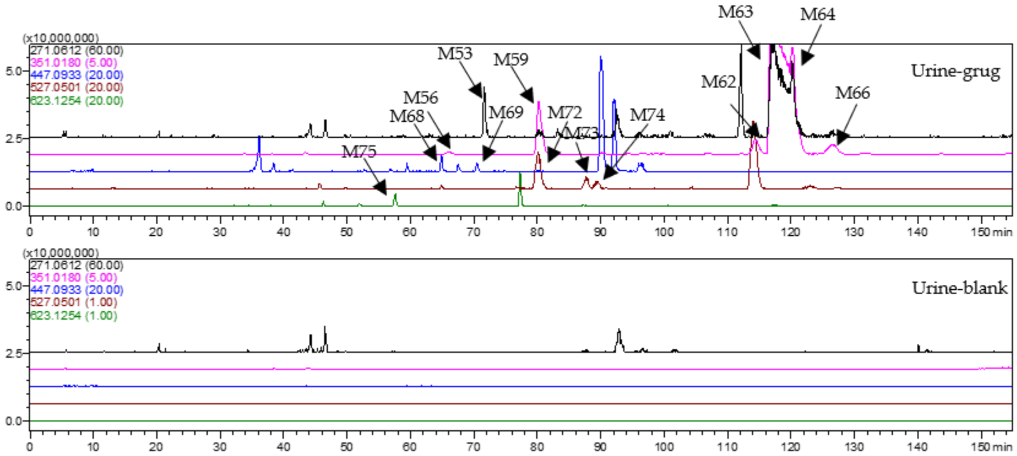

Figure S18. EICs of *m*/*z* 271.0612, *m*/*z* 351.0180, *m*/*z* 447.0933, *m*/*z* 527.0501 and *m*/*z* 623.1254 in drug urine and blank urine in ESI− mode.

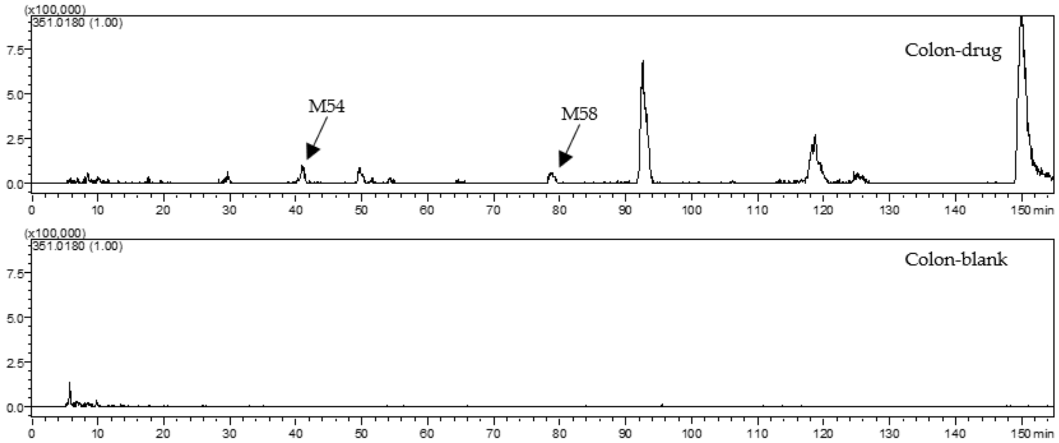

Figure S19. EICs of *m*/*z* 351.0180 in drug colon and blank colon in ESI− mode.

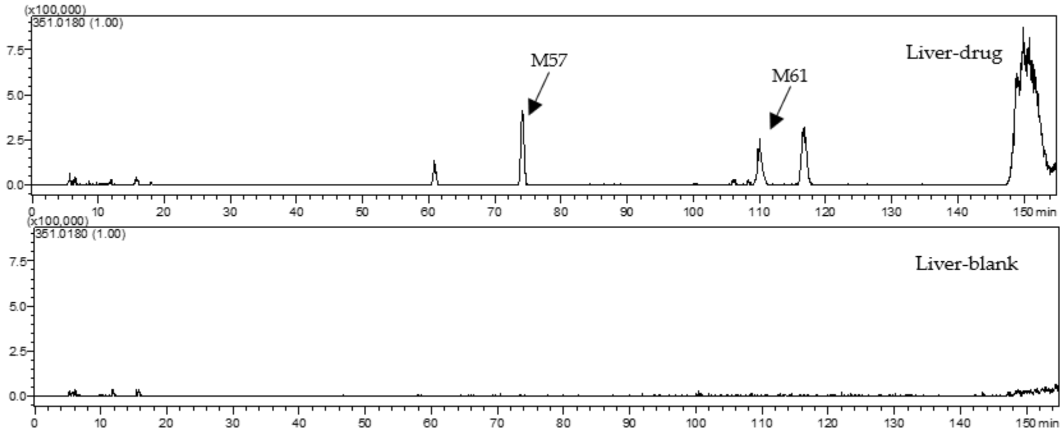

Figure S20. EICs of *m*/*z* 351.0180 in drug liver and blank liver in ESI− mode.

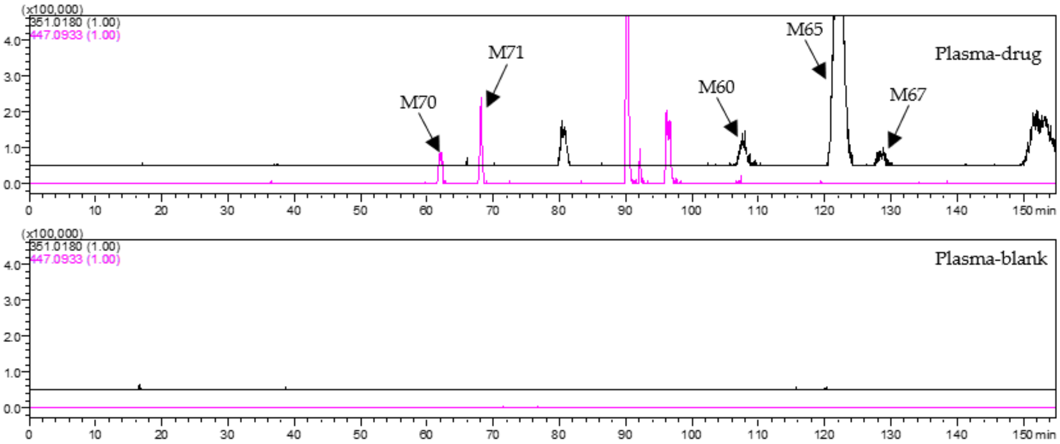

Figure S21. EICs of *m*/*z* 351.0180 and 447.0933 in drug plasma and blank plasma in ESI− mode.

(6) metabolites (**M76–M107**) having the skeleton of hydroxylated medicarpin

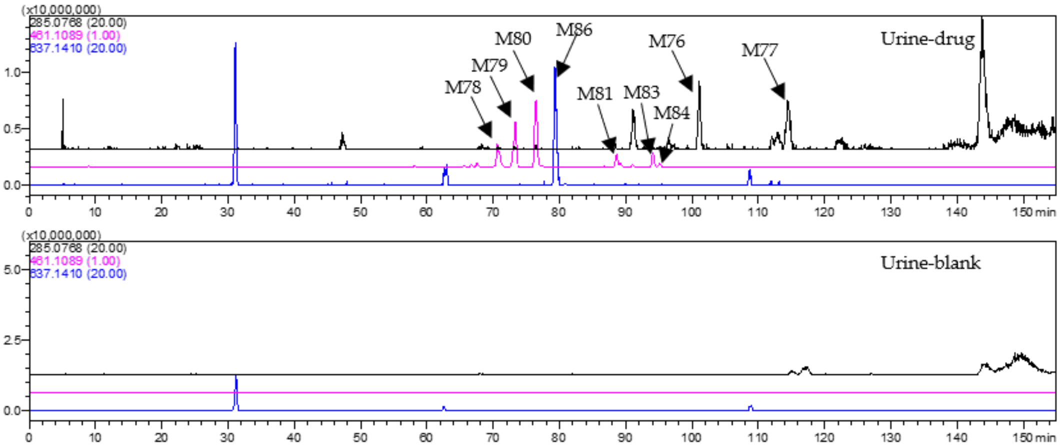

Figure S22. EICs of *m*/*z* 285.0768, *m*/*z* 461.1089 and *m*/*z* 637.1410 in drug urine and blank urine in ESI− mode.

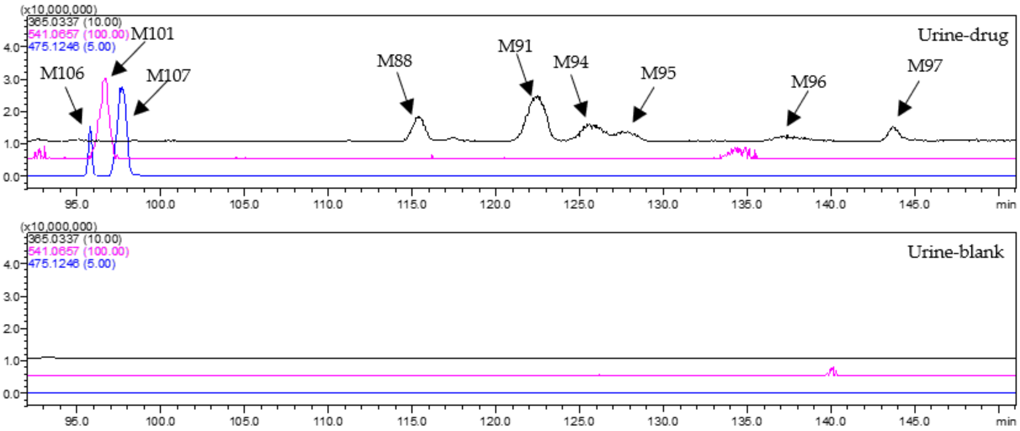

Figure S23. EICs of *m*/*z* 365.0337, *m*/*z* 541.0657 and *m*/*z* 475.1246 in drug urine and blank urine in ESI− mode.

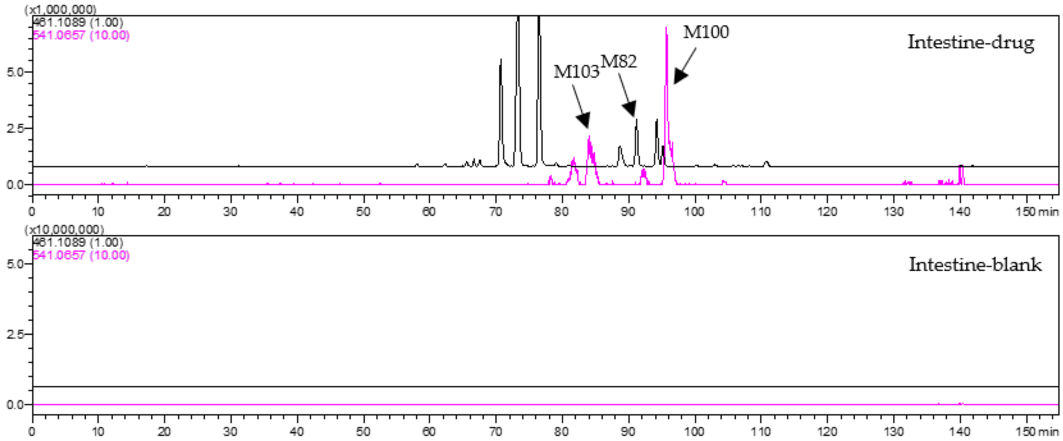

Figure S24. EICs of *m/z* 461.1089 and 541.0657 in drug intestine and blank intestine in ESI− mode.

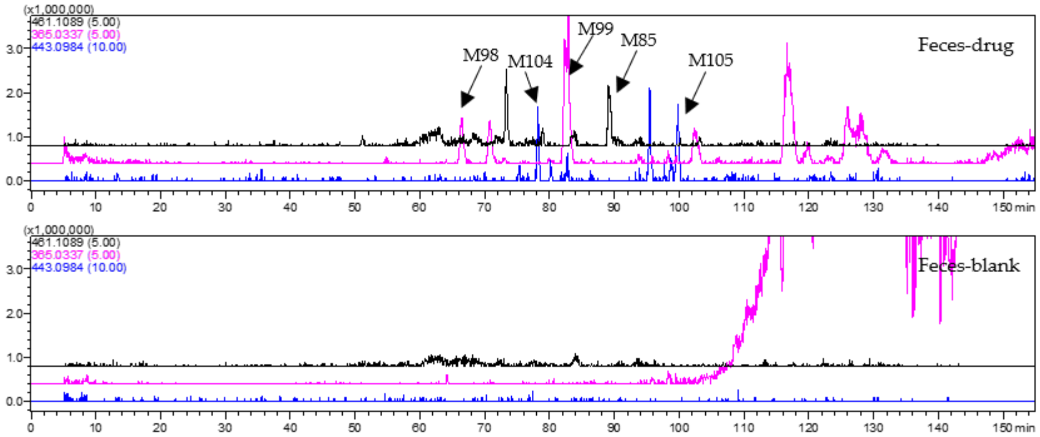

Figure S25. EICs of *m/z* 461.1089, *m/z* 365.0337 and *m/z* 443.0984 in drug feces and blank feces in ESI− mode.

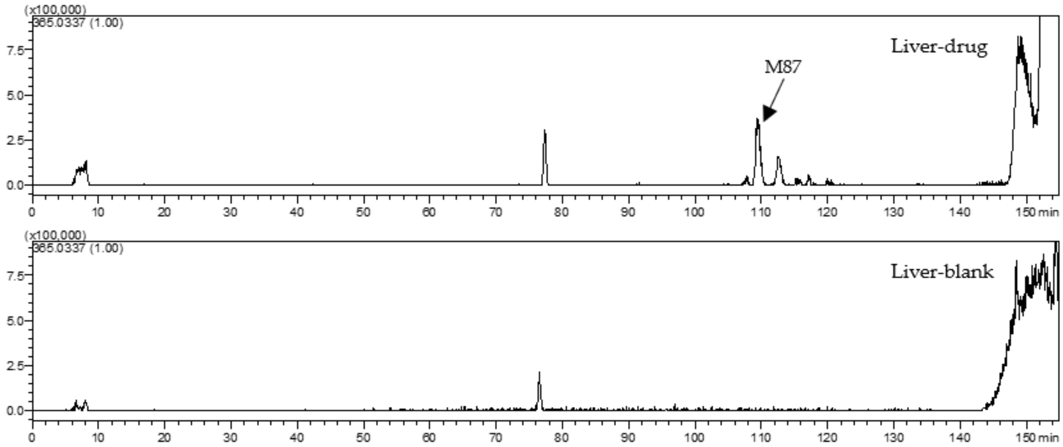

Figure S26. EICs of *m/z* 365.0337 in drug liver and blank liver in ESI− mode.

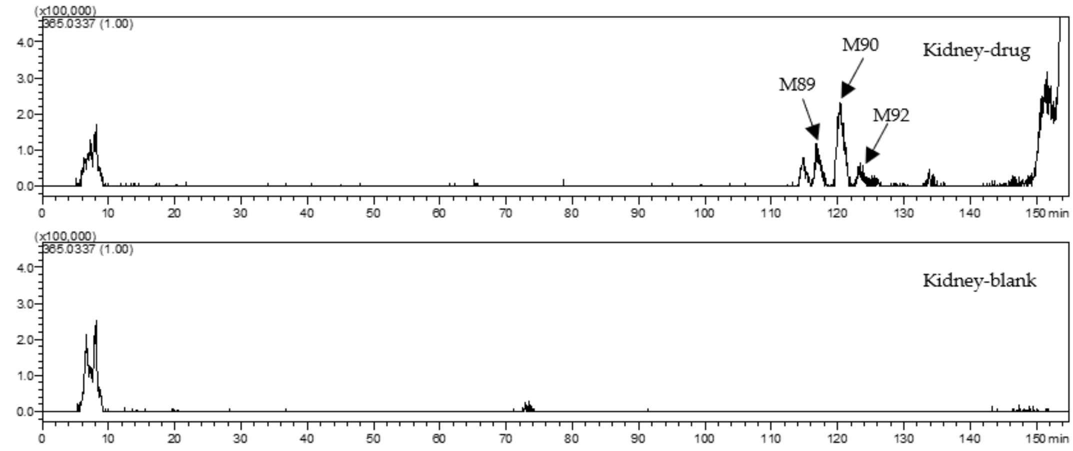

Figure S27. EICs of *m/z* 365.0337 in drug kidney and blank kidney in ESI− mode.

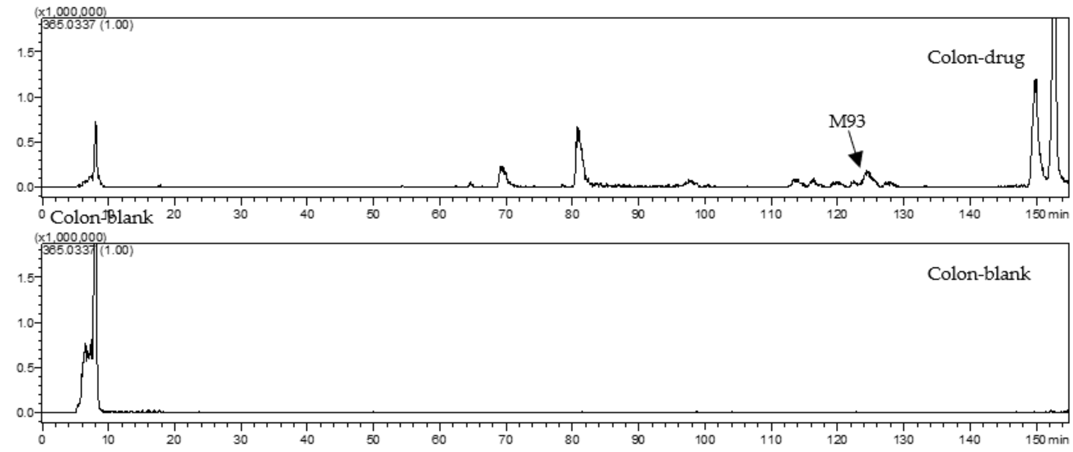

Figure S28. EICs of *m/z* 365.0337 in drug colon and blank colon in ESI− mode.

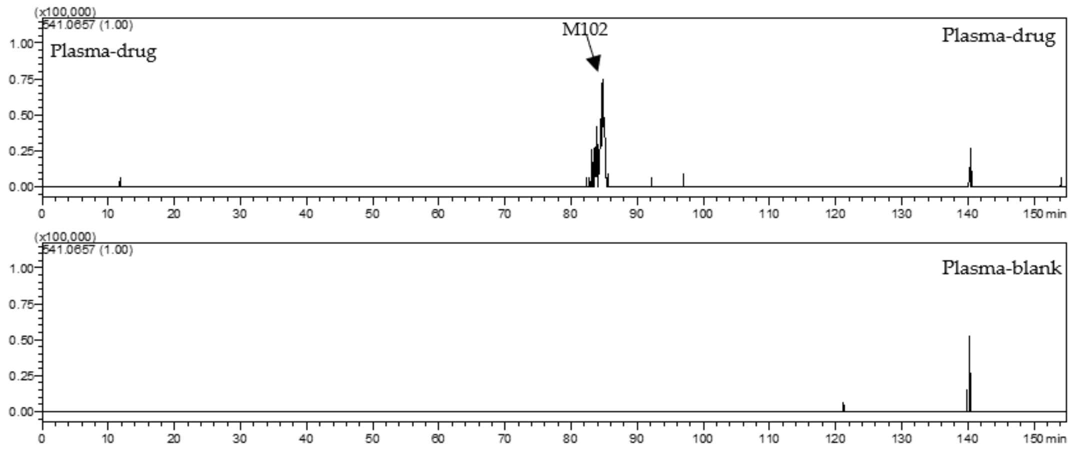

Figure S29. EICs of *m/z* 541.0657 in drug plasma and blank plasma in ESI− mode.

(7) metabolites (**M108–M138**) having the skeleton of hydrogenated and hydroxylated medicarpin

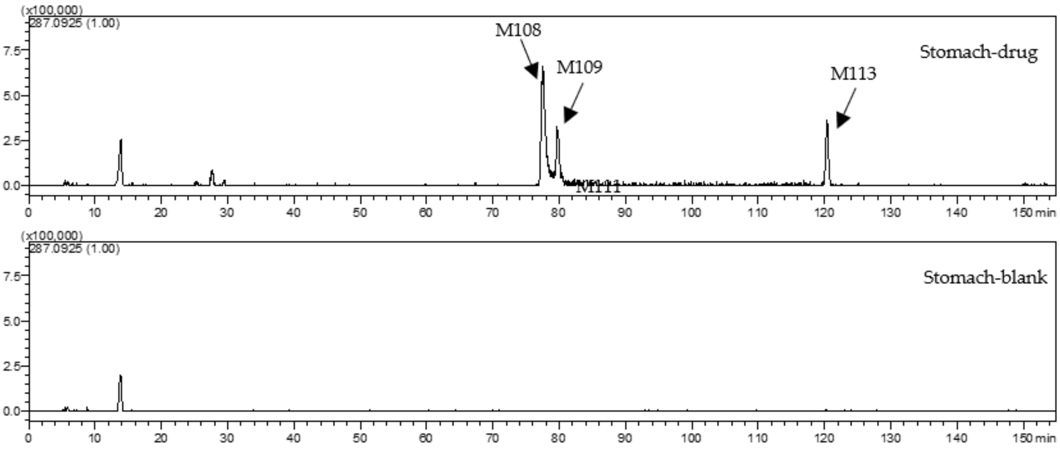

Figure S30. EICs of *m*/*z* 287.0925 in drug stomach and blank stomach in ESI− mode.

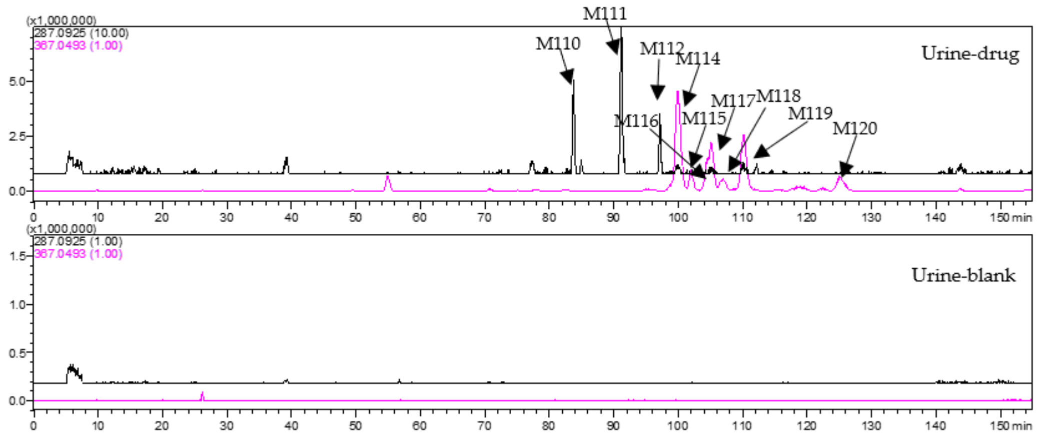

Figure S31. EICs of *m*/*z* 287.0925 and 367.0925 in drug urine and blank urine in ESI− mode.

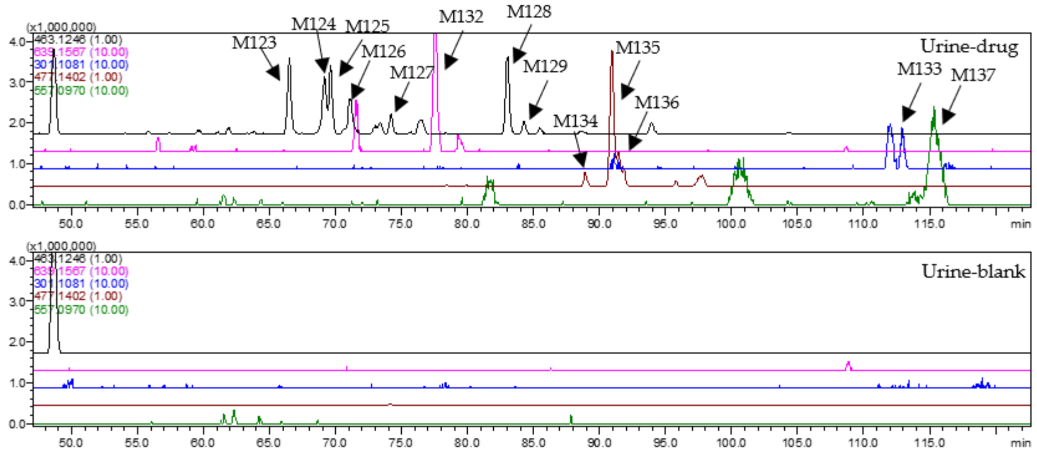

Figure S32. EICs of *m*/*z* 463.1246, *m*/*z* 639.1567, *m*/*z* 301.1081, *m*/*z* 477.1402 and *m*/*z* 557.0970 in drug urine and blank urine in ESI− mode.

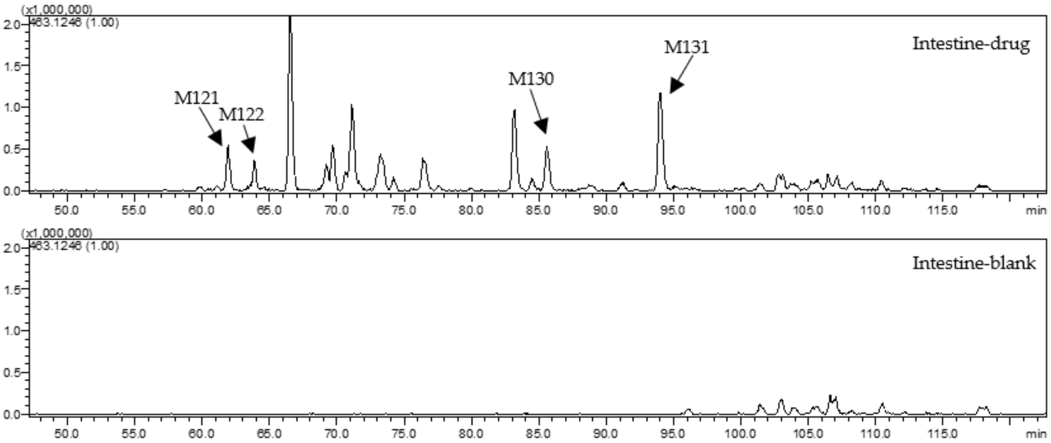

Figure S33. EICs of *m*/*z* 463.1246 in drug intestine and blank intestine in ESI− mode.

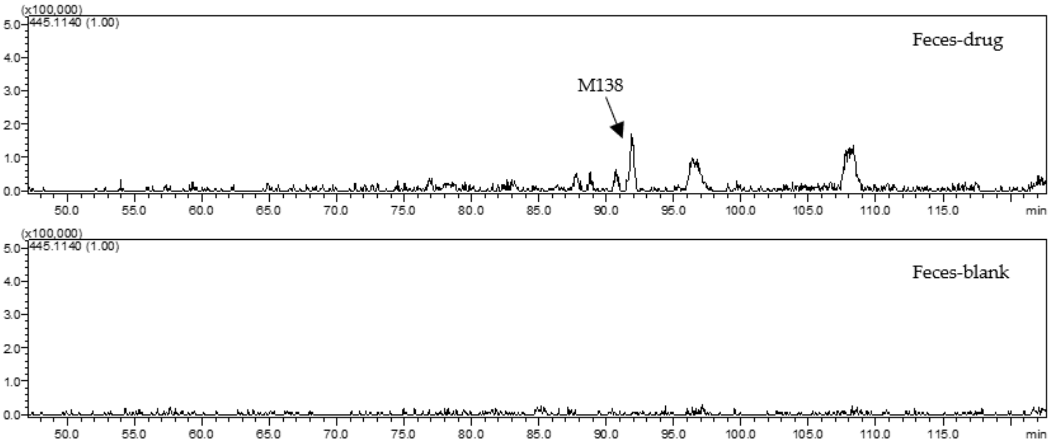

Figure S34. EICs of *m*/*z* 445.1140 in drug feces and blank feces in ESI− mode.

(8) metabolites (**M139–M149**) having the skeleton of demethylated, hydrogenated and hydroxylated medicarpin

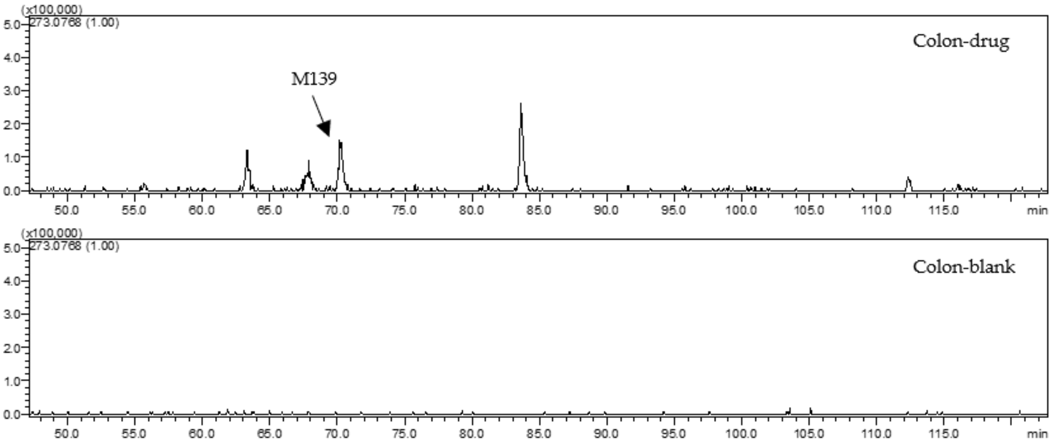

Figure S35. EICs of *m*/*z* 273.0768 in drug colon and blank colon in ESI− mode.

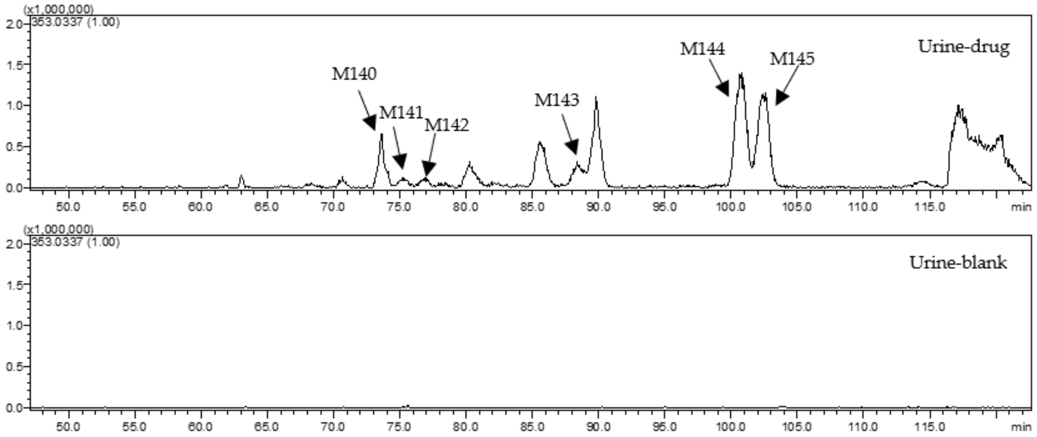

Figure S36. EICs of *m/z* 353.0337 in drug urine and blank urine in ESI− mode.

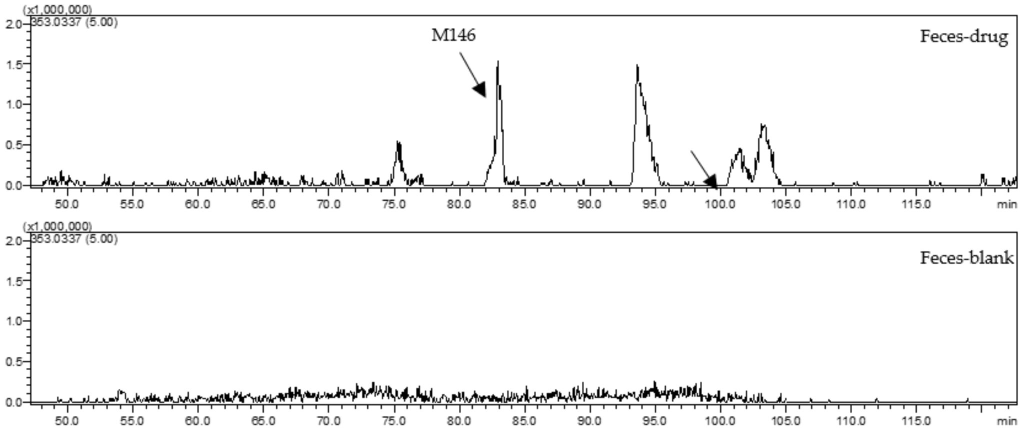

Figure S37. EICs of *m/z* 353.0337 in drug feces and blank feces in ESI− mode.

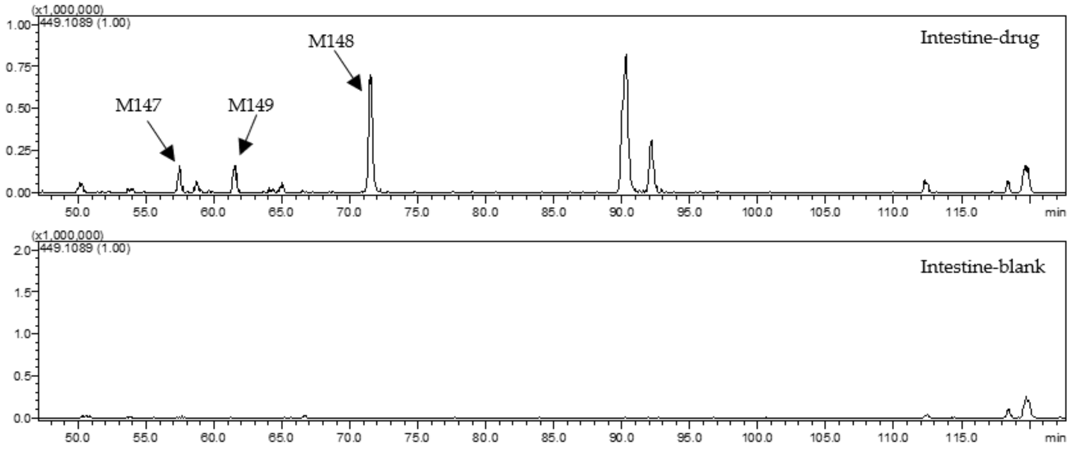

Figure S38. EICs of *m/z* 449.1089 in drug intestine and blank intestine in ESI− mode.

(9) metabolites (**M150–M156**) having the skeleton of demethylated and dihydroxylated medicarpin

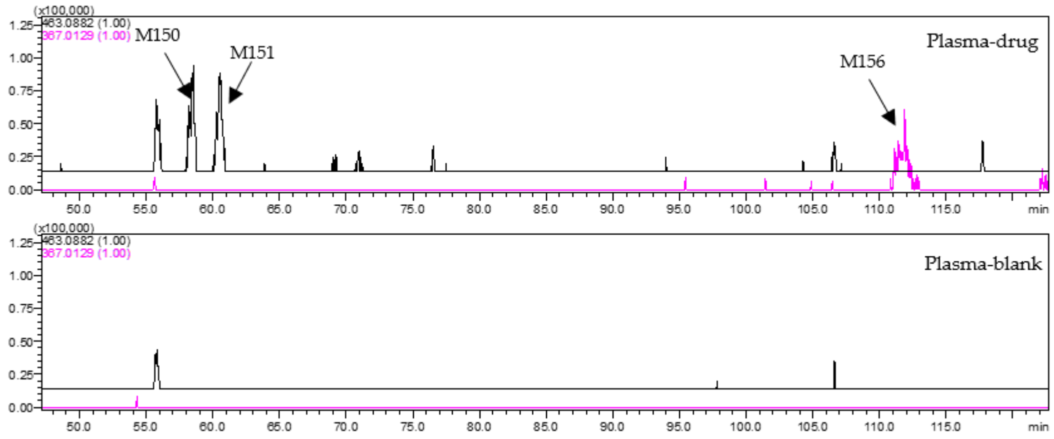

Figure S39. EICs of *m/z* 463.0882 and 367.0129 in drug plasma and blank plasma in ESI− mode.

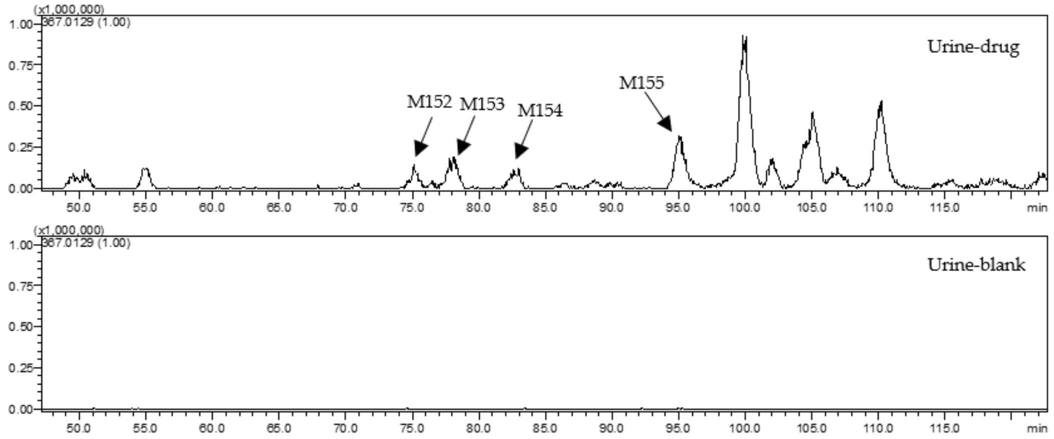

Figure S40. EICs of *m/z* 367.0129 in drug urine and blank urine in ESI− mode.

(10) metabolites (**M157–M161**) having the skeleton of dihydroxylated medicarpin

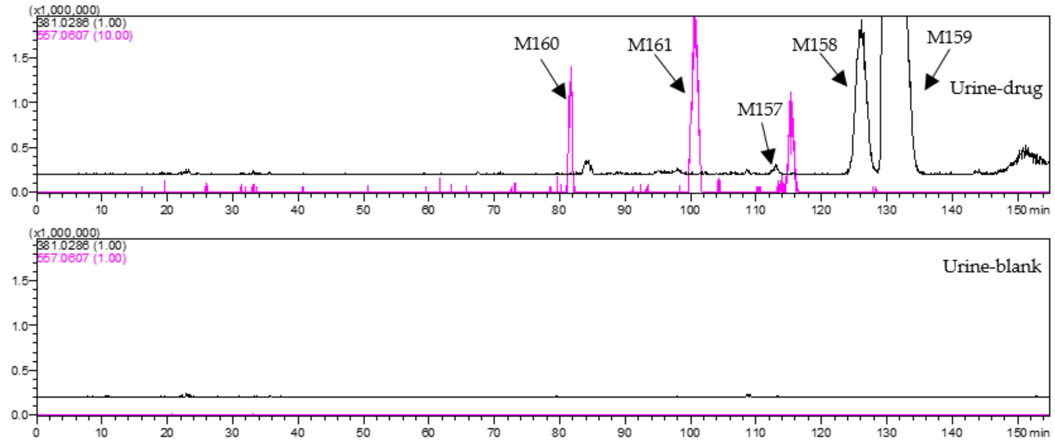

Figure S41. EICs of *m/z* 381.0286 and 557.0607 in drug urine and blank urine in ESI− mode.

(11) metabolite (**M162**) having the skeleton of hydrogenated and dihydroxylated medicarpin

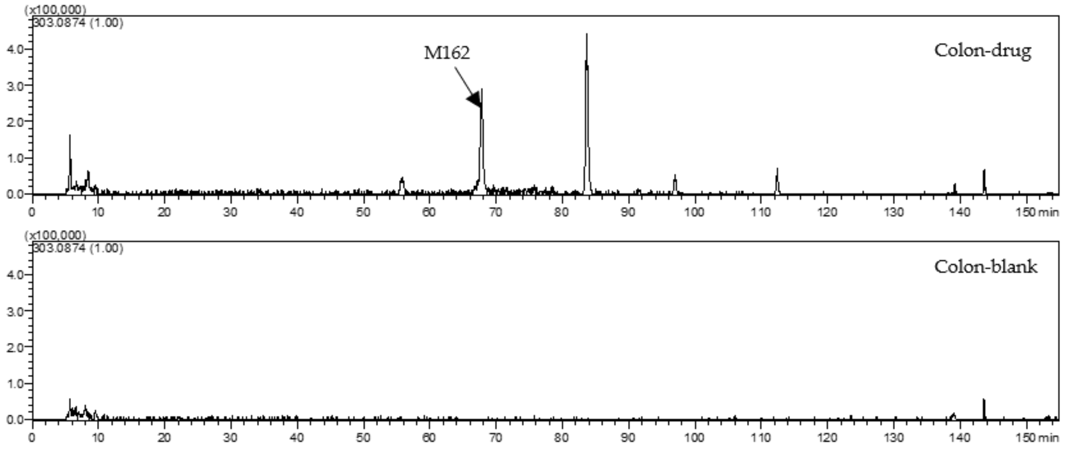

Figure S42. EICs of *m/z* 303.0874 in drug colon and blank colon in ESI− mode.

(12) metabolites (**M163–M164**) having the skeleton of hydrogenated and trihydroxylated medicarpin

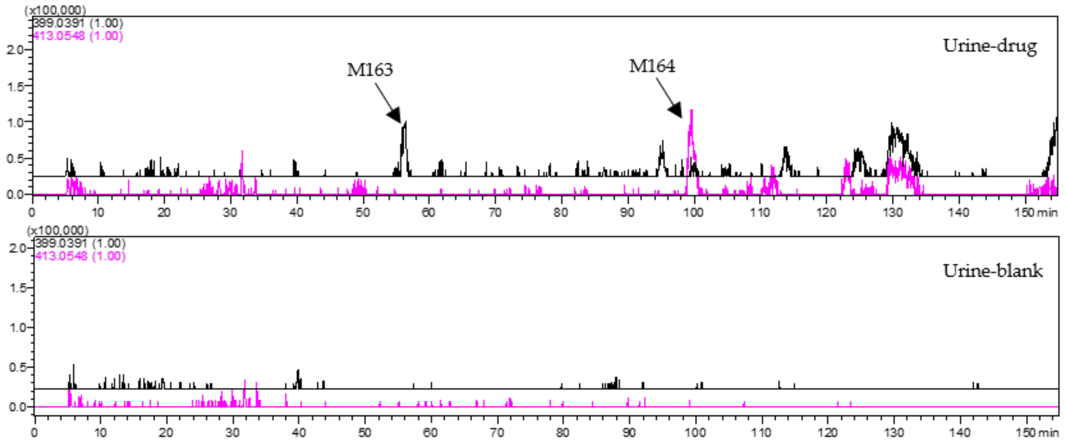

Figure S43. EICs of *m/z* 399.0391 and 557.0607 in drug urine and blank urine in ESI− mode.

(13) metabolite (**M165**) has the skeleton of demethylated, hydrogenated and dihydroxylated medicarpin

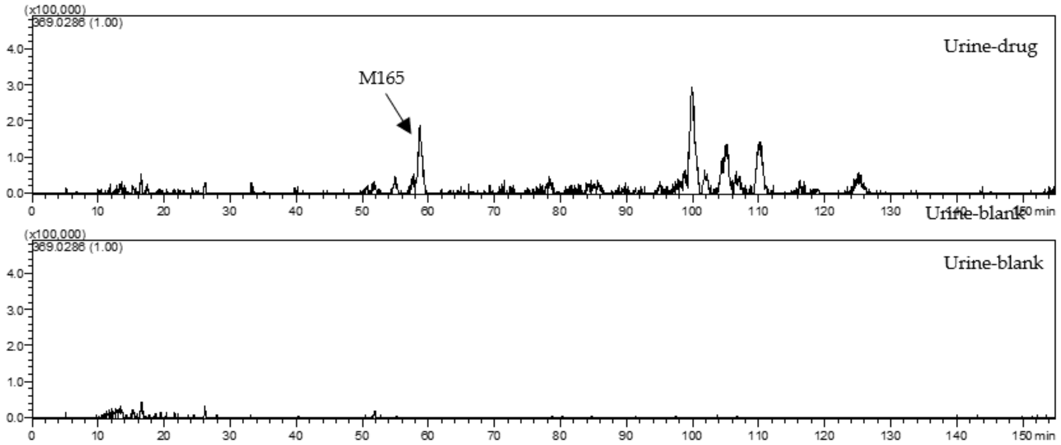

Figure S44. EICs of *m/z* 369.0286 in drug urine and blank urine in ESI− mod.

2. The MS spectra of M4, M10, M37, M112, M114, M115, M144 and M159 (Figure S45–S52)

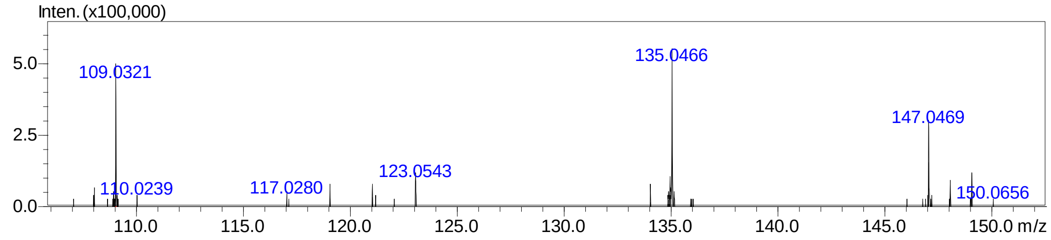

Figure S45. The MS² spectrum of **M4**.

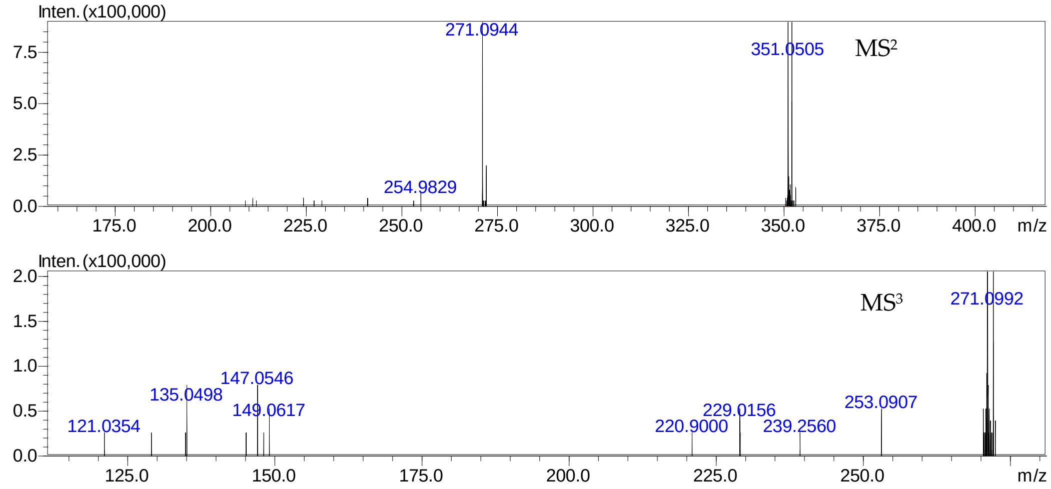

Figure S46. The MS² and MS³ spectra of **M10**.

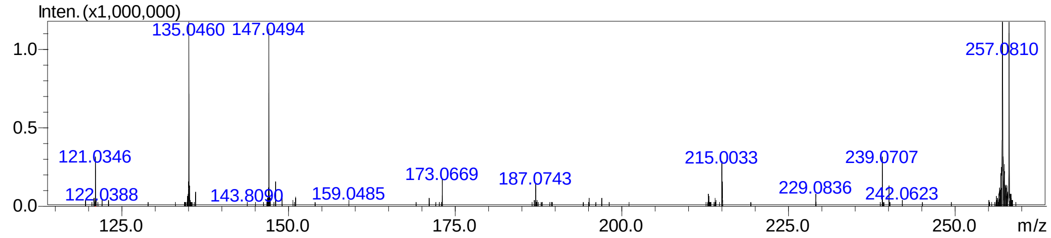

Figure S47. The MS² spectrum of **M37**.

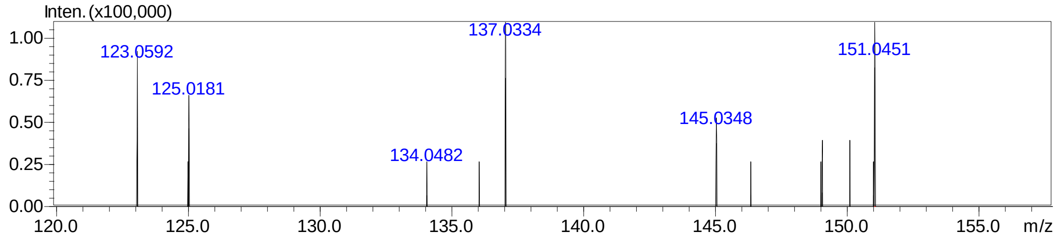

Figure S48. The MS² spectra of **M112**.

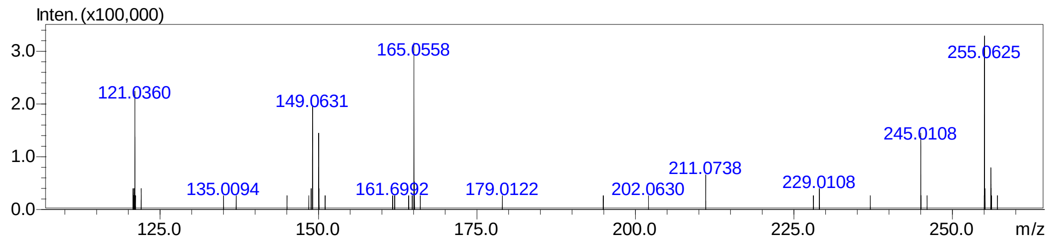

Figure S49. The MS² spectrum of **M114**.

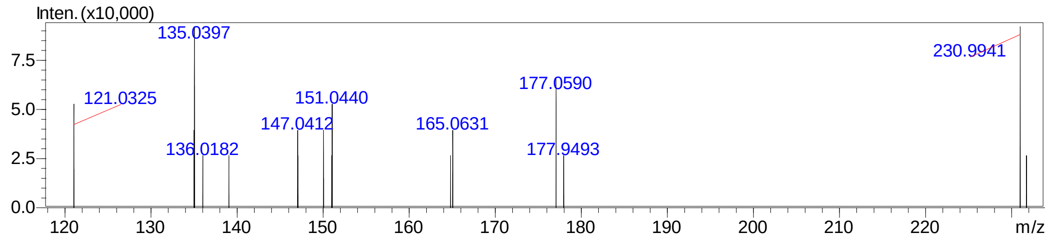

Figure S50. The MS² spectrum of **M115**

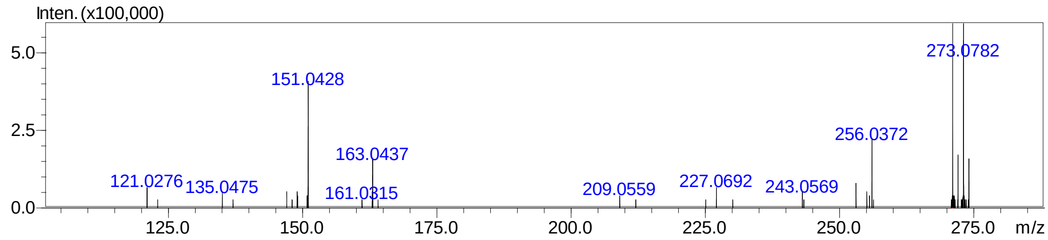

Figure S51. The MS² spectrum of **M144**.

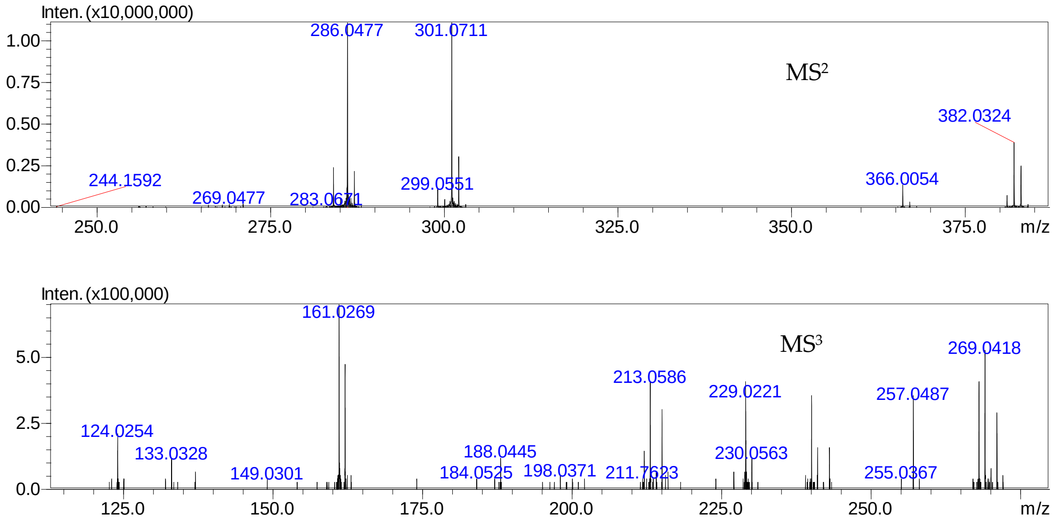

Figure S52. The MS² and MS³ spectra of **M159**.
